# freqpcr: Estimation of population allele frequency using qPCR ΔΔCq measures from bulk samples

**DOI:** 10.1111/1755-0998.13554

**Published:** 2021-12-09

**Authors:** Masaaki Sudo, Masahiro Osakabe

**Affiliations:** ^1^ Division of Fruit Tree and Tea Pest Control Research Institute for Plant Protection NARO: Kanaya Tea Research Station Shimada Japan; ^2^ Laboratory of Ecological Information Graduate School of Agriculture Kyoto University Kyoto Japan

**Keywords:** confidence interval, group testing, maximum‐likelihood estimation, R language, real‐time polymerase chain reaction

## Abstract

PCR techniques, both quantitative (qPCR) and nonquantitative, have been used to estimate the frequency of a specific allele in a population. However, the labour required to sample numerous individuals and subsequently handle each sample renders the quantification of rare mutations (e.g., pesticide resistance gene mutations at the early stages of resistance development) challenging. Meanwhile, pooling DNA from multiple individuals as a “bulk sample” combined with qPCR may reduce handling costs. The qPCR output for a bulk sample, however, contains uncertainty owing to variations in DNA yields from each individual, in addition to measurement errors. In this study, we have developed a statistical model to estimate the frequency of the specific allele and its confidence interval when the sample allele frequencies are obtained in the form of ΔΔCq in the qPCR analyses on multiple bulk samples collected from a population. We assumed a gamma distribution as the individual DNA yield and developed an R package for parameter estimation, which was verified using real DNA samples from acaricide‐resistant spider mites, as well as a numerical simulation. Our model resulted in unbiased point estimates of the allele frequency compared with simple averaging of the ΔΔCq values. The confidence intervals suggest that dividing the bulk samples into more parts will improve precision if the total number of individuals is equal; however, if the cost of PCR analysis is higher than that of sampling, increasing the total number and pooling them into a few bulk samples may also yield comparable precision.

## 1 INTRODUCTION

Estimating the frequency of specific alleles in populations is a technique ubiquitous in population genetics, molecular ecology, evolutionary biology and their areas of application. Indices of genetic differentiation between populations are defined based on allele frequency measurement for one or more loci, on which phylogenetic analyses have been established (Takezaki & Nei, 1996; Wright, 1965). Allele frequencies fluctuate between generations due to adaptation or genetic drift. In evolutionary genetics, multilocus and/or time‐series data of single nucleotide polymorphisms (SNPs) are used to detect natural selection (Nielsen, 2005), adaptive introgression (Hedrick, 2013) and historical events such as population bottlenecks (Luikart et al., 1999; Schwartz et al., 2007).

There are also growing demands for allele monitoring in biological conservation and industrial sectors such as food production. Using SNP data, conservation biologists have assessed the parameters associated with the local extinction risk, such as effective population size and migration rates (Leitwein et al., 2020). The detection and frequency estimation of DNA polymorphism are major techniques to monitor the invasion and population establishment of invasive species and/or a species that is close to and may hybridize with a cultured species (Dias et al., 2008; Zaccara et al., 2021). Field monitoring has also been performed to detect resistance genes of arthropod pests to pesticides and genetically modified insecticidal plants, such as *Bt* crops (Andow & Alstad, 1998; Sonoda et al., 2017). Although entomologists have traditionally estimated resistance allele frequencies via bioassays, molecular diagnostics have been developed in accordance with the recent development of genome‐wide association studies on resistance genes (Donnelly et al., 2016; ffrench‐Constant, 2013; Samayoa et al., 2015; Snoeck et al., 2019; Sugimoto et al., 2020; Toda et al., 2017).

While allele frequency measurement using genetic diagnostic techniques is becoming widespread, finite sample size still brings uncertainty to estimate population allele frequencies. If the target population is sufficiently large, the alleles are distributed randomly in the population and the genotype is known for each individual organism, simple binomial assumption provides us with the point estimate and its confidence interval (Fung & Keenan, 2014). However, individual DNA analysis, imposing the cost of sample preprocessing, may not be feasible for large numbers of individuals. It becomes a problem particularly when the frequency estimation of a rare (<1%) mutation is required, which is often the case, for example, in the early phase of resistance development.

**FIGURE 1:**
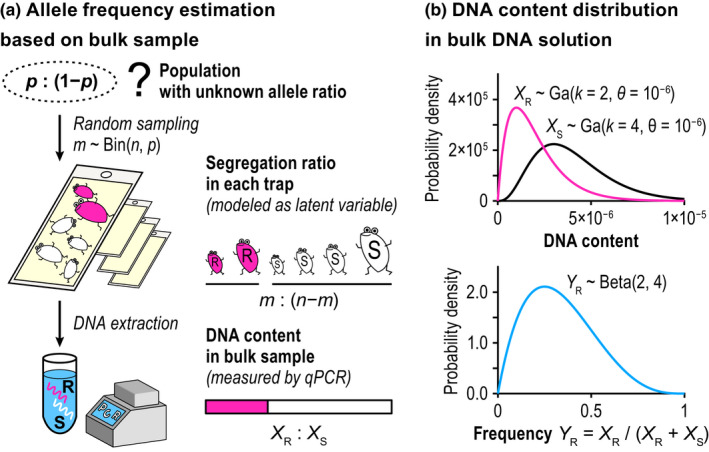
Population allele frequency estimation based on bulk samples. (a) Each bulk sample is obtained by collecting *n* (haploid) individuals, of which *m* have the resistant (R) and *n *−* m* have the susceptible (S) alleles. The DNA content in the bulk sample does not strictly correspond to *m*: (*n *−* m*) because they reflect differences in DNA yields among individuals. (b) The allelic DNA amounts in the bulk sample are assumed to independently follow the gamma distribution, whereas R frequency follows the beta distribution

Pooling multiple individual samples and processing a single DNA extract (i.e., the use of a “bulk sample”) may reduce the required time and cost associated with handling multiple samples (Figure 1a). In coordination with statistical methods such as group testing, it can guarantee precision and accuracy of the population allele frequency estimation at a certain level (Rode et al., 2018; Yamamura & Hino, 2007).

qPCR, based on real‐time PCR, has been proposed for the point estimation of the allele frequencies from bulk samples. To quantify the relative amount of two alleles on a specific locus in a bulk sample, Germer et al. (2000) adopted an index called ΔCq, which is usually defined as the difference between the cycles of quantification (Cq) in the real‐time PCR on equal volumes of dispensed DNA solution. In their study, ΔCq was defined as the difference of the Cq values for two solutions dispensed from a bulk sample, each amplified with the primer sets corresponding to a specific allele on the target locus. This approach required a calibration curve because there was no guarantee that the amplification efficiencies using the two primer sets were equal.

**FIGURE 2:**
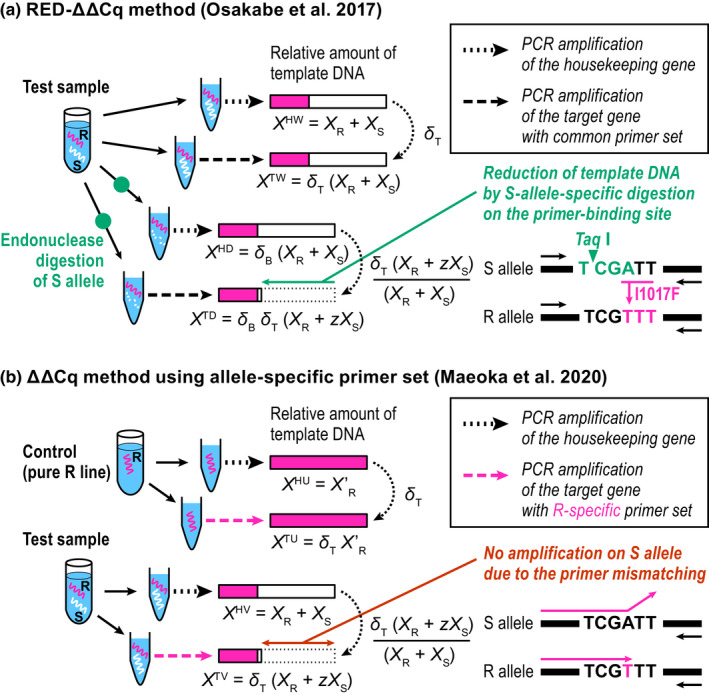
Schema for estimation of target mutation frequency in a bulk DNA sample using qPCR. (a) The restriction enzyme digestion (RED)‐ΔΔCq analysis and (b) the ΔΔCq analysis using an R‐specific primer set. The lengths of the bars correspond to the relative DNA quantities of R and S alleles (*X*_R_ and *X*_S_). In either method, the frequency of *X*_R_ in a test sample is quantified as $X_{R}+zX_{S}$ ($≅X_{R}$) measured on the target gene, divided by *X*_R_ + *X*_S_ measured on a housekeeping gene in the sample. As the copy numbers may differ between genes, the relative content *δ*_T_ is also quantified using a control or undigested portion of the sample

Osakabe et al. (2017) developed a method called “RED‐ΔΔCq method” (RED, restriction enzyme digestion) for the genetic diagnostics of resistance in the two‐spotted spider mite, *Tetranychus urticae* Koch (Acari: Tetranychidae), to the acaricide etoxazole, which is conferred by an amino acid substitution in chitin synthase 1 (*CHS1*; I1017F) (Van Leeuwen et al., 2010). For the relative quantification of the resistant allele to the susceptible allele in a bulk sample, the RED‐ΔΔCq method used a nonspecific primer set to amplify both alleles on the resistance‐associated locus. At the same time, half of the dispensed solutions had been digested beforehand with restriction endonucleases (Figure 2a). The restriction site was designed to recognize only the susceptible allele on the target locus; thereby, the ratio of the resistant to the (resistant + susceptible) alleles was compared. The changes in DNA concentration before and after the digestion were corrected using the Cq values measured for a housekeeping gene as an internal reference of DNA quantities for each treatment level, following a common method of qPCR known as the ΔΔCq method (Livak & Schmittgen, 2001). In the etoxazole‐R diagnostics of Osakabe et al. (2017), glyceraldehyde‐3‐phosphate dehydrogenase (*GAPDH*) was used as the reference gene because the parallelism in the PCR amplification efficiencies of *CHS1* and *GAPDH* was kept over the DNA concentration range. Although the calibration curve and two specific primers sets used by Germer et al. (2000) are no longer needed, the RED‐ΔΔCq method still depends on the availability of the restriction enzyme. Maeoka et al. (2020) later demonstrated a general ΔΔCq method without restriction enzyme treatment for measuring allele frequency if a single specific primer set was designed to amplify one of the two alleles (Figure 2b).

However, the measurement of population allele frequencies via the qPCR methods still lacks a group testing framework for interval estimation. Although the ΔΔCq measure in Maeoka et al. (2020) and Osakabe et al. (2017) gives the point estimate for the allele frequency in each bulk sample, the error structure for the DNA amounts intrinsic in the bulk sample has not been modelled. Unlike the individual PCR or sequencing analyses where the genotype is determined for each individual, population allele frequency estimated from bulk samples results in a wider range of confidence intervals than that associated with binomial distribution because the DNA yield of each individual is probably not constant (Rode et al., 2018). A possible solution to regulate the error is modelling explicitly the individual DNA yields, which will follow a certain probability distribution, in addition to the binomial assumption on the number of individuals contained in each bulk sample (Figure 1b).

In this study, we propose a statistical method to obtain the confidence interval of the population allele frequency using ΔΔCq‐based qPCR analyses for multiple bulk samples taken from a population. A random error structure is introduced to approximate the relative abundance of the two alleles and their ratio in the bulk DNA sample. This structure is decomposed into two parts: the relationship between the population allele frequency and the amounts of the template DNA in the bulk sample consisting of a certain number of randomly collected individuals. Another component is the error specific to the (RED)‐ΔΔCq measures in the qPCR analyses. We then develop a maximum‐likelihood estimation procedure to estimate the frequency of a specific allele and its confidence interval in the population, which was evaluated with real DNA samples from the etoxazole‐resistant spider mites and numerical simulations. Notably, an R package source is available online (https://github.com/sudoms/freqpcr).

## 2 MODEL

### 2.1 Approximation of allele quantities contained in a bulk DNA sample

When DNA is directly extracted from the whole body of a living organism, the DNA yield is roughly proportional to its body weight (Chen et al., 2010). For insects, the intrapopulation frequency distribution of body weight is often approximated using a unimodal and right‐skewed continuous distribution, typically a lognormal or gamma distribution (Knapp, 2016; May, 1976). Although Gouws et al. (2011) suggested that body weights are distributed lognormally in many nonsocial insect species, it is difficult to distinguish which distribution a real population obeys. The two distributions are considered interchangeable when the sample size is not large (Kundu & Manglick, 2005; Wiens, 1999).

In this study, we adopted a gamma, rather than lognormal, distribution to approximate the DNA amount per individual organism because the sum and proportion of independent gamma distributions have closed forms under certain conditions (Mitchell et al., 2015). Using Equation 1, let *X* (*X* ≥ 0) be the DNA yield per single locus per individual:

(1) $$\text{Ga}\left(X|k,\theta \right)=\frac{1}{\Gamma \left(k\right)}{\left(\frac{1}{\theta }\right)}^{k}X^{k‐1}\exp\left(‐\frac{X}{\theta }\right)$$

where $\Gamma \left(\cdot \right)$ denotes the gamma function. The parameters *k* and *θ* (*k*, *θ* > 0) are the shape and scale parameters of the gamma distribution, respectively. The mean is given by *kθ*.

Using Equation 1, let us consider the amounts of allelic DNA in the sample extracted from multiple individuals at once, hereafter referred to as a “bulk sample.” Table 1 lists the variables and parameters of the model structure. For simplicity, we model the case of haploidy in the main text. Appendix S1 describes the approximated formulation for diploids. Let us assume that we have *n* insects, of which *m* (*m* = 0, 1, ..., *n*) are the genotypes resistant to an insecticide (hereafter denoted by R). The rest n‐m carries S, the susceptible allele. When we capture insects from a wild population, the size of *n* is obvious, but *m* is usually unknown (Figure 1a). Assuming random sampling from an infinite population with the R allele at frequency *p*, *m* follows a binomial distribution (Equation 2):

(2) $$\text{Bin}\left(m|n,p\right)=\frac{n!}{m!\left(n‐m\right)!}p^{m}{\left(1‐p\right)}^{n‐m}.$$

**TABLE 1:** Description of variables and parameters

Symbol	Description	Range	Arguments in the numerical experiment and the R package
p	Frequency of the R (resistant) allele in a population	0≤p≤1	P
$X_{S}$, $X_{R}$	Amounts of DNA belonging to S (susceptible) or R alleles included in a bulk sample	$X_{S}\geq 0$, $X_{R}\geq 0$	—
$Y_{R}$	The observed frequency of R in the bulk sample, defined as $X_{R}/\left(X_{R}+X_{S}\right)$	$0\leq Y_{R}\leq 1$	—
k, θ	Shape and scale parameters of the gamma distribution $\text{Ga}\left(k,\theta \right)$	k>0, θ>0	K
N	Number of bulk samples taken from a population	N∈N	ntrap
n, $n_{h}$	Number of individuals constituting the (*h*th) bulk sample	n∈N	npertrap (fixed in the numerical experiment)
$\sum_{h=1}^{N}n_{h}$	Total sample size	—	ntotal
m, $m_{h}$	Number of R individuals included in the (*h*th) bulk sample	0≤m∈Z≤n	m (as an internal variable)
**qPCR‐related variables and parameters**			
η	Per‐cycle efficiency in the PCR amplification (as 1+η)	η>0	EPCR
$X_{\Theta }$	The termination threshold of the amplification in real‐time PCR	$X_{\Theta }>0$	Fixed 1 in the package
τ	Cq value: the number of PCR amplification cycles before termination	τ∈R	$\tau_{h}^{\text{TW}}$: target0, $\tau_{h}^{\text{TD}}$: target1, $\tau_{h}^{\text{HW}}$: housek0, $\tau_{h}^{\text{HD}}$: housek1
$\delta_{T}$	Relative content of the target gene to the internal reference (housekeeping gene)	$\delta_{T}>0$	targetScale
$\delta_{B}$	(In the RED‐ΔΔCq method) the locus‐independent change rate of the template DNA quantity accompanying the restriction enzyme treatment	$\delta_{T}>0$	baseChange
z	(In the RED‐ΔΔCq method) residual rate of restriction enzyme digestion, or (in general ΔΔCq analyses) portion of the off‐target allele amplified in the PCR	z>0	zeroAmount
$\varepsilon_{c}$	Cq measurement error (standard deviation)	$\varepsilon_{c}>0$	sdMeasure

When the bulk sample contains at least one resistant individual, $X_{R}=\sum_{i=1}^{m}X_{i}$ denotes the total R content where $X_{i}$ is the individual DNA yield. If there is no systematic error in the efficiency of DNA extraction between the genotypes and if $X_{i}$ obeys the gamma distribution of Equation 1, then $X_{R}$ follows the gamma distribution with the shape parameter mk and scale parameter θ based on the reproducible property (Figure 1b). Conversely, the amount of the S allele is denoted by $X_{S}=\sum_{i=1}^{n}X_{i}$, which follows the gamma distribution with $\left(n‐m\right)k$ and θ.

$$X_{R}∼\text{Ga}\left(mk,\theta \right),$$

(3) $$X_{S}∼\text{Ga}\left(\left(n‐m\right)k,\theta \right).$$

When $X_{R}$ and $X_{S}$ independently follow gamma distributions with the same scale parameter, the observed allele frequency $Y_{R}=X_{R}/\left(X_{S}+X_{R}\right)$ follows a beta distribution with the shape parameters mk and $\left(n‐m\right)k$:

(4) $$\text{Beta}\left(Y_{R}|mk,\left(n‐m\right)k\right)=\frac{Y_{R}^{mk‐1}{\left(1‐Y_{R}\right)}^{\left(n‐m\right)k‐1}}{B\left(mk,\left(n‐m\right)k\right)},$$

where $B\left(\cdot \right)$ is a beta function. This error structure was originally developed by Sudo et al. (2021) to model allele frequencies measured via quantitative sequencing, in which the gamma distributions were used to approximate the yield variations due to body weight plus post‐mortem DNA degradation on a trap. Of note, the gamma distribution has recently been used to approximate the DNA release of aquatic animals and DNA abundance in water (Fukaya et al., 2021).

### 2.2 Relative quantification of allelic DNA by real‐time PCR

#### 2.2.1 Allele frequency estimation from a single bulk sample: RED‐ΔΔCq method

In the RED‐ΔΔCq method of Osakabe et al. (2017), the control was prepared as an intact bulk sample containing total DNA ($=X_{R}+X_{S}$) on the target locus. The sample in question was the same DNA extract, whereas it was digested with restriction endonucleases prior to qPCR analysis (Figure 2a). The restriction site is designed to recognize the S allele on the target locus to ensure that the operation digests the major part of S (denoted by 1‐z: z is a small yet positive variable giving the residual rate). Consequently, we obtained the template amount $X_{R}+zX_{S}$ at the target locus after digestion. To calibrate the template DNA amounts, samples before and after digestion were also amplified using the primer set for a housekeeping gene as an internal reference.

Taken together, the single bulk sample results in a quartet of Cq measurements differentiating at the target loci (resistance‐associated and housekeeping genes) × restriction enzyme digestion (undigested and digested). We can then formulate the allele frequencies by letting $X^{\text{HW}}$ and $X^{\text{TW}}$ represent the total amounts of template DNA at the housekeeping (H) and target (T) loci, respectively, included in the sample without digestion, the state denoted by W.

(5) $$\begin{array}{ccc} X^{\text{HW}} & = & X_{R}+X_{S},\\ X^{\text{TW}} & = & \delta_{T}\left(X_{R}+X_{S}\right). \end{array}$$

The coefficient $\delta_{T}$ ($\delta_{T}>0$) provides the relative content of the target gene to the housekeeping gene in genomic DNA (the difference in the DNA extraction efficiencies is also included). After digestion (state D), $X^{\text{HD}}$ and $X^{\text{TD}}$ denote the DNA amounts at the H and T loci, respectively:

(6) $$\begin{array}{ccc} X^{\text{HD}} & = & \delta_{B}\left(X_{R}+X_{S}\right),\\ X^{\text{TD}} & = & \delta_{B}\delta_{T}\left(X_{R}+zX_{S}\right). \end{array}$$

The common coefficient $\delta_{B}$ ($\delta_{B}>0$) provides the rate of certain locus‐independent changes in the quantities of template DNA accompanying the restriction enzyme treatment.

As a result of qPCR, the Cq quartet, $\tau^{\text{HW}}$, $\tau^{\text{TW}}$, $\tau^{\text{HD}}$ and $\tau^{\text{TD}}$ were obtained as:

$$\begin{array}{cc} \tau^{\text{HW}} & =\frac{\text{ln}\left(X_{\Theta }\right)‐\text{ln}\left(X_{R}+X_{S}\right)}{\text{ln}\left(1+\eta \right)}+\varepsilon_{c},\\ \tau^{\text{TW}} & =\frac{\text{ln}\left(X_{\Theta }\right)‐\text{ln}\delta_{T}‐\text{ln}\left(X_{R}+X_{S}\right)}{\text{ln}\left(1+\eta \right)}+\varepsilon_{c}, \end{array}$$

(7) $$\begin{array}{cc} \tau^{\text{HD}} & =\frac{\text{ln}\left(X_{\Theta }\right)‐\text{ln}\delta_{B}‐\text{ln}\left(X_{R}+X_{S}\right)}{\text{ln}\left(1+\eta \right)}+\varepsilon_{c},\\ \tau^{\text{TD}} & =\frac{\text{ln}\left(X_{\Theta }\right)‐\text{ln}\delta_{B}‐\text{ln}\delta_{T}‐\text{ln}\left(X_{R}+zX_{S}\right)}{\text{ln}\left(1+\eta \right)}+\varepsilon_{c}. \end{array}$$

Here, 1+η (η>0) and $X_{\Theta }$ denote the amplification efficiency per PCR cycle and its threshold, respectively. According to Livak and Schmittgen (2001), we assume an ideal amplification, where $X_{\Theta }$ is set within the early exponential amplification phase. The actual Cq data contain measurement errors in addition to uncertainty due to experimental operations, such as sample dispensation or PCR amplification. We express these using the common error term $\varepsilon_{c}∼N\left(0,\sigma_{c}^{2}\right)$, following the normal distribution of mean = 0 and variance = $\sigma_{c}^{2}$ in the scale of raw Cq values. The validity of this error structure is verified later.

The two ΔCq values were then defined for the undigested and digested samples, as $\Delta \tau^{W}=\tau^{\text{TW}}‐\tau^{\text{HW}}$ and $\Delta \tau^{D}=\tau^{\text{TD}}‐\tau^{\text{HD}}$, respectively. Their ΔΔCq are:

(8) $$\Delta \Delta \tau =\Delta \tau^{D}‐\Delta \tau^{W}=‐\frac{\ln\left(\frac{X_{R}+zX_{S}}{X_{R}+X_{S}}\right)}{\ln\left(1+\eta \right)}+\varepsilon ,\;\;\;\;\;\varepsilon ∼N\left(0,4\sigma_{c}^{2}\right).$$

From Equation 8, the expected value of $\left(X_{R}+zX_{S}\right)/\left(X_{R}+X_{S}\right)$ is calculated as ${\left(1+\eta \right)}^{‐\Delta \Delta \tau }$. The coefficients $\delta_{B}$ and $\delta_{T}$ in Equations 5 and 6 vanished by subtracting the Cq values and ΔCq values, respectively.

The point estimate of the resistance allele frequency, ${\hat{Y}}_{R}$, is defined as $X_{R}/\left(X_{R}+X_{S}\right)$ for each bulk sample. When z is much smaller than ${\hat{Y}}_{R}$, the quantity $\left(X_{R}+zX_{S}\right)/\left(X_{R}+X_{S}\right)={\hat{Y}}_{R}+z\left(1‐{\hat{Y}}_{R}\right)$ itself can approximate the frequency, which will be the case with enough digestion time before qPCR. However, use of the point estimate may introduce a problem in that the size of ${\hat{Y}}_{R}$ often exceeds 1 when the R frequency is high, and a larger error exists in the Cq measurement (see Experiment 2).

Although the value of 1+η may vary on the primer sets, both target and housekeeping loci share the same amplification efficiency in Equation 7, because practical PCR protocols were designed to be 1+η≅2. We can also approximately cancel the effect of heterogeneous amplification efficiencies by fitting the $\delta_{T}$ size of the sample sets with known allele ratios (Experiment 1).

#### 2.2.2 Measurement of ΔΔCq using allele‐specific primer sets

Although the RED‐ΔΔCq method enabled us to measure allele frequency from the bulk sample, enzyme availability is a prerequisite to digest the S‐allele‐specific restriction site at the target locus. A longer digestion period (3 h) was also required to quantify etoxazole resistance in the protocol by Osakabe et al. (2017).

Maeoka et al. (2020) demonstrated that a general ΔΔCq method without restriction enzyme treatment could be used for allele‐frequency measurement if a specific primer set were to be designed to amplify only the R allele at the target locus. Similar to the RED‐ΔΔCq method, DNA samples with unknown mixing ratios were dispensed and amplified using primer sets corresponding to T and H loci, respectively. Unlike the RED‐ΔΔCq method, the control sample was not taken from the test sample solution but was prepared as a DNA solution containing 100% R, hereafter denoted as U (= pUre R line) (Figure 2b).

$X^{\text{HU}}$ and $X^{\text{TU}}$ then denote the template DNA quantities in the control sample:

(9) $$\begin{array}{cc} X^{\text{HU}} & =X_{R}^{\prime },\\ X^{\text{TU}} & =\delta_{T}X_{R}^{\prime }. \end{array}$$

Although the definition of $\delta_{T}$ is the same as in Equation 5, the quantity is denoted by $X_{R}^{\prime }$ instead of $X_{S}+X_{R}$ as it no longer originates from the R portion of the test sample itself (i.e., not internal).

For the test sample (denoted as V), the template DNA quantities amplified at the housekeeping ($X^{\text{HV}}$) and target ($X^{\text{TV}}$) loci are expressed as follows:

(10) $$\begin{array}{cc} X^{\text{HV}} & =X_{R}+X_{S},\\ X^{\text{TV}} & =\delta_{T}\left(X_{R}+zX_{S}\right). \end{array}$$

In the PCR process of the modified ΔΔCq method, the small positive number *z* provides the template quantity of S, which is nonspecifically amplified even with the R‐specific primer set. As the primer set for the housekeeping gene was nonspecific, $X^{\text{HV}}$ was fully amplified. Assuming that all four template DNAs are amplified with efficiency 1+η, we define the two ΔCq values as $\Delta \tau^{U}=\tau^{\text{TU}}‐\tau^{\text{HU}}$ and $\Delta \tau^{V}=\tau^{\text{TV}}‐\tau^{\text{HV}}$. Finally, their ΔΔCq values are $\Delta \Delta \tau =\Delta \tau^{V}‐\Delta \tau^{U}$, yielding a formula identical to Equation 8.

### 2.3 Simultaneous interval estimation of allele frequency and experimental parameters based on qPCR over multiple bulk samples

Finally, we consider the likelihood model to obtain the interval estimate of the allele frequency based on the (RED‐)ΔΔCq analysis over multiple bulk samples. Assume that the population has the R allele at frequency p from which N bulk samples are taken. The *h*th sample (h=1,2,3,...,N) consists of $n_{h}$ haploid individuals, of which *m*_h_ are resistant mutants. As shown in Equation 7, the Cq values (denoted as $\tau_{h}^{\text{HW}}$, $\tau_{h}^{\text{TW}}$, $\tau_{h}^{\text{HD}}$ and $\tau_{h}^{\text{TD}}$ for each bulk sample) are determined not only by the DNA quantities, denoted as $X_{h,R}$ and $X_{h,S}$, but also by parameters such as $\delta_{T}$ or $\sigma_{c}^{2}$ accompanying the experimental operation. We can simultaneously estimate these if we have multiple bulk samples, for which the likelihood function of obtaining the Cq values under the parameters is defined.

We propose the joint likelihood for the two ΔCq values, $\Delta \tau_{h}^{W}=\tau_{h}^{\text{TW}}‐\tau_{h}^{\text{HW}}$ and $\Delta \tau_{h}^{D}=\tau_{h}^{\text{TD}}‐\tau_{h}^{\text{HD}}$, for the convenience of numerical calculation:

$$\Delta \tau_{h}^{W}∼N\left(‐\frac{\text{ln}\delta_{T}}{\text{ln}\left(1+\eta \right)},2\sigma_{c}^{2}\right),$$

(11) $$\Delta \tau_{h}^{D}∼N\left(‐\frac{\text{ln}\delta_{T}\;\text{+ ln}\left(\frac{X_{h,R}+zX_{h,S}}{X_{h,R}+X_{h,S}}\right)}{\text{ln}\left(1+\eta \right)},2\sigma_{c}^{2}\right).$$

Although Equation 11 is defined for the RED‐ΔΔCq method, it can also be applied to the ΔΔCq method of Maeoka et al. (2020) by substituting $\Delta \tau_{h}^{W}$ and $\Delta \tau_{h}^{D}$ with $\Delta \tau_{h}^{U}=\tau_{h}^{\text{TU}}‐\tau_{h}^{\text{HU}}$ and $\Delta \tau_{h}^{V}=\tau_{h}^{\text{TV}}‐\tau_{h}^{\text{HV}}$, respectively.

#### 2.3.1 Formulation of likelihood based on the gamma or beta distribution

Using the relationship between $m_{h}$, $n_{h}$ and p in Equation 2, we proceed to the likelihood function defined as the probability of observing the set of $\Delta \tau_{h}^{W}$ and $\Delta \tau_{h}^{D}$ under the given values of p, $n_{h}$ and other experimental parameters. In Equation 11, $\Delta \tau_{h}^{W}$ is not affected by the R: S ratio in the bulk sample; it is only affected by the experimental parameters, $\delta_{T}$, η and $\sigma_{c}^{2}$. In addition, by taking the differences, there is no need to estimate $X_{\Theta }$ and $\delta_{B}$ appearing in Equation 7. Moreover, cancellation of $\delta_{B}$ also ensures that we can apply the model of Equation 11 to the general ΔΔCq method of Equations 9 and 10.

Conversely, we must consider the amount of DNA in the bulk sample to calculate the probability of obtaining $\Delta \tau_{h}^{D}$. When the size of *m*_h_ is specified under the binomial assumption, the quantities of DNA in the *h*th bulk sample, $X_{h,\text{R|}m_{h}}$ and $X_{h,\text{S|}m_{h}}$, can independently take any positive values following the gamma distribution of Equation 3, and their proportions $Y_{h,\text{R|}m_{h}}=X_{h,\text{R|}m_{h}}/\left(X_{h,\text{R|}m_{h}}+X_{h,\text{S|}m_{h}}\right)$ are $\text{Beta}\left(m_{h}k,\left(n_{h}‐m_{h}\right)k\right)$ as shown in Equation 4. If the sample contains only S or R, then $X_{h,\text{R|}m_{h}=0}=0$ or $X_{h,\text{S|}m_{h}=n_{h}}=0$ is guaranteed.

The likelihood function for the observed ΔCq values on the *h*th bulk sample $L_{h}$ is defined as follows:

$$L_{h}=P\left(\Delta \tau_{h}^{W}|\delta_{T},\eta ,\sigma_{c}^{2}\right)\sum_{m_{h}=0}^{n_{h}}\left[\text{Bin}\left(m_{h}|n_{h},p\right)P\left(\Delta \tau_{h}^{D}|m_{h},\delta_{T},z,\eta ,\sigma_{c}^{2}\right)\right],$$

(12) $$P\left(\Delta \tau_{h}^{D}|m_{h},\delta_{T},z,\eta ,\sigma_{c}^{2}\right)=\left\{\begin{array}{cc} N\left(‐\frac{\text{ln}\left(z\delta_{T}\right)}{\text{ln}\left(1+\eta \right)},2\sigma_{c}^{2}\right) & \left(m_{h}=0\right)\\ \begin{array}{ccc} \psi_{G} & \text{or} & \psi_{B} \end{array} & \left(m_{h}=1,2,...,n_{h}‐1\right)\\ N\left(‐\frac{\text{ln}\delta_{T}}{\text{ln}\left(1+\eta \right)},2\sigma_{c}^{2}\right) & \left(m_{h}=n_{h}\right) \end{array}\right..$$

In Equation 12, $\psi_{G}$ or $\psi_{B}$ denotes the probability of obtaining $\Delta \tau_{h}^{D}$ under the template DNA quantities of $X_{h,\text{R|}m_{h}}=r$ and $X_{h,\text{S|}m_{h}}=s$ if we model the two quantities by a gamma distribution, or if we formularize their mixing ratio by the single beta distribution, respectively. We must consider not only the possible cases of *m*_h_, but also the entire range of the DNA amounts. If we use the gamma distributions, for every case $m_{h}=1,2,...,n_{h}‐1$, we need to calculate the double integration for $\psi_{G}$ under the whole region of $X_{h,\text{R|}m_{h}}=r$ and $X_{h,\text{S|}m_{h}}=s$ for the interval $\left\{D:0\leq r<\infty ,0\leq s<\infty \right\}$.

(13) $$\psi_{G}=\iint_{D}N\left(‐\frac{\text{ln}\delta_{T}\;\text{+ ln}\left(\frac{r+zs}{r+s}\right)}{\text{ln}\left(1+\eta \right)},2\sigma_{c}^{2}\right)\text{Ga}\left(r|m_{h}k,\theta \right)\text{Ga}\left(s|\left(n_{h}‐m_{h}\right)k,\theta \right)drds.$$

The common scale parameter of the gamma distributions, θ, is not identifiable from the data, although we can substitute arbitrary values θ=1 for it because Equation 13 can also be expressed using $\text{Ga}\left(\left(r/\theta \right)|m_{h}k,1\right)$ and $\text{Ga}\left(\left(s/\theta \right)|\left(n_{h}‐m_{h}\right)k,1\right)$. Thereafter, θ is cancelled in $\left(r+zs\right)/\left(r+s\right)$ and has no effect on the parameter set that optimizes $\psi_{G}$.

Since the computational burden for the double integration is large, we simplified the likelihood model with the beta distribution. By introducing $y=r/\left(r+s\right)$, the probability of obtaining $\Delta \tau_{h}^{D}$ is replaced with $\psi_{B}$ defined as follows:

(14) $$\psi_{B}=\int_{0}^{1}N\left(‐\frac{\text{ln}\delta_{T}\;\text{+ ln}\left(z+y\left(1‐z\right)\right)}{\text{ln}\left(1+\eta \right)},2\sigma_{c}^{2}\right)\text{Beta}\left(y|m_{h}k,\left(n_{h}‐m_{h}\right)k\right)dy.$$

We provide an R function “freqpcr()” to estimate the parameters p, k, $\delta_{T}$ and $\sigma_{c}$ simultaneously when the set of Cq measurements ($\tau_{h}^{\text{HW}}$, $\tau_{h}^{\text{TW}}$, $\tau_{h}^{\text{HD}}$ and $\tau_{h}^{\text{TD}}$) and $n_{h}$ are given for each of the N bulk samples. This function did not work when we measured the Cq values over only one bulk sample because it is expected to estimate up to four parameters while the data is input as two difference values ($\Delta \tau_{h}^{W}$ and $\Delta \tau_{h}^{D}$). The default is freqpcr(…, beta = TRUE), where the beta distribution model of Equation 14 was used instead of gamma. Regardless of the algorithms, the asymptotic confidence intervals are calculated using the inverse of the Hessian matrix evaluated at the last iteration. The functions nlm() of R and cubintegrate() in the R package “cubature” (Narasimhan et al., 2019) are used for the iterative optimization and the (double) integration, respectively.

### 2.4 Identification of auxiliary parameters using DNA samples with known allele‐mixing ratios

The likelihood introduced above ensures that we can estimate the sizes of p and k together with other experimental parameters if we have conducted a (RED‐)ΔΔCq analysis on multiple bulk samples. However, the size of *z*, the residue rate of the S allele, is not identified and must be specified as a fixed parameter. The amplification efficiency, η, is estimated in theory over the iterative calculation of Equation 11, but it is the only parameter appearing in the denominators. Simultaneous estimation sometimes fails when η is set as unknown.

Therefore, the experimenter should identify the sizes of these auxiliary parameters. To estimate their plausible sizes, one can conduct (RED‐)ΔΔCq analysis using DNA solutions with known allele ratios; for instance, DNA can be extracted from each of the pure breeding lines of S and R and mix the solutions at multiple ratios, or make a dilution series of R by S. As the ratio of $X_{R}$ to $X_{S}$ is strictly fixed, Equation 7 is directly applicable to express the relationship between DNA quantities and the four Cq measurements. The R functions knownqpcr() and knownqpcr_unpaired() appearing in the package provide the maximum‐likelihood estimation for $\delta_{B}$, $\delta_{T}$, $\sigma_{c}$, z and η. These values can be used as fixed parameters in the freqpcr() function. The “knownqpcr_unpaired” function was developed to handle incomplete data (i.e., the observations of $\tau^{\text{HW}}$, $\tau^{\text{TW}}$, $\tau^{\text{HD}}$ and $\tau^{\text{TD}}$ have different data lengths). If the four Cq measures are available for all samples, then “knownqpcr” is used.

Another objective of the analysis with known‐ratio samples is to test the homoscedasticity of the qPCR data at the scale of Cq measures. Regarding the relationship between the etoxazole‐R allele frequency in *Tetranychus urticae* and the corresponding 2^−ΔΔCq^ measures (the approximate point estimate of the frequency), Osakabe et al. (2017) demonstrated linearity using a sample series of DNA with multiple mixing ratios on CHS1 (I1017F). In the next section, we recycled the same data to compare whether the Cq measurements in the RED‐ΔΔCq analysis obey the homoscedasticity in the scale of ΔΔCq or (1 + *η*)^−ΔΔCq^.

## 3 MATERIALS AND METHODS

### 3.1 Experiment 1: Estimation of auxiliary parameters and verification of homoscedasticity in Cq measurements based on mite DNA samples with known allele‐mixing ratios

#### 3.1.1 Experimental setup

In the experiment by Osakabe et al. (2017), the resistant mite strain (SoOm1‐etoR strain) originated from a field population collected in Omaezaki City, Shizuoka, Japan (34.7°N, 138.1°E) in January 2012. The susceptible strain was obtained from Kyoyu Agri Co., Ltd (Kyoyu‐S strain). For each strain, two pairs of females and males were used separately. Each pair was allowed to mate and oviposit on a kidney bean leaf square (2 × 2 cm) for 4 days. The mites were then confirmed to be homozygous on the CHS1 locus using sequence analysis. Genomic DNA extracted from the offspring of each pair was used for qPCR analysis. For each pair, the DNA extracts were prepared twice, each of which was a mixture from 50 adult females homogenized together, that is, four extracts (replicates) for each strain.

To verify the validity of the RED‐ΔΔCq method, qPCR analysis was performed with heterogeneous DNA solutions with 10 mixing ratios of $X_{R}/\left(X_{R}+X_{S}\right)$ = {0, 0.001, 0.005, 0.01, 0.05, 0.1, 0.25, 0.5, 0.75, 1}. The net DNA concentration of each mixed solution was adjusted to 1 ng µl^−1^, from which 15 ng was dispensed into each of the two tubes. Only one was digested with the restriction enzymes before qPCR. For digestion, the samples were treated with a mixture of two enzymes, *MluC* I (10 units) and *Taq* ^α^I (20 units; New England BioLabs), at 37°C for 3 h, followed by incubation at 65°C for 3 h, which is due to the polymorphism of the CHS1 loci; the 1017 codon of *Tetranychus urticae* displays ATT (Kyoyu‐S strain) or TTT (SoOm1‐etoR) sequences, whereas the upstream 1016 codon displays a synonymous TCG or TCA independent of the strains (Van Leeuwen et al., 2012). Therefore, we need to digest both TCGATT (underline shows the restriction site of *Taq^α^* I) and TCAATT (*MluC* I) to diminish the entire S allele.

qPCR analysis using the intercalator method was performed using the LightCycler Nano System (Roche Diagnostics) with SYBR Fast qPCR Mix (Takara) as described previously (Osakabe et al., 2017). The primer sets were tu03*CHS1* (forward: 5ʹ‐GGCACTGCTTCATCCACAAG‐3ʹ and reverse: 5ʹ‐GTGTTCCCCAAGTAACAACGTTC‐3ʹ) and tu25*GAPDH* (forward: 5ʹ‐GCACCAAGTGCTAAAGCATGGAG‐3ʹ and reverse: 5ʹ‐GAACTGGAACACGGAAAGCCATAC‐3ʹ) for the resistance‐associated and housekeeping loci, respectively.

#### 3.1.2 Statistical analysis

The maximum likelihood of $\delta_{B}$, $\delta_{T}$, $\sigma_{c}$, z and η was conducted with the “knownqpcr_unpaired” function of the freqpcr package (version 0.3.5). The raw Cq data are available as Appendix S3 (ESM) 1 along with a step‐by‐step guide for statistical analyses (ESM 2). Due to the limitation of the handling capacity of the thermal cycler, qPCR analysis was not conducted on undigested samples of the nine mixing ratios other than $X_{R}/\left(X_{R}+X_{S}\right)=1$ (i.e., pure R solution). Thus, in each replicate, Osakabe et al. (2017) used the observed $\Delta \tau^{W}$ value when the ratio = 1 for other ratios to calculate the conventional ΔΔCq indices. As we have shown in Equation 7, this operation does not affect the point estimates of *p*, although the size of the Cq measurement error ($\sigma_{c}$) will be underestimated if we recycle the observed Cq value multiple times.

Regarding the relationship between the true mixing ratio and the RED‐ΔΔCq measures in the sample, the linearity was analysed using a linear model via the function “lm” running on R version 3.6.1 (R Core Team, 2019), where the response variables were put into the model at the scale of Cq or (1 + *η*)^−ΔΔCq^. Based on the linear models, we tested heteroscedasticity using the Breusch‐Pagan test via the bptest() function of the R library “lmtest” (Hothorn et al., 2019).

### 3.2 Experiment 2: Evaluation of the simultaneous estimation method with randomly generated data

Since the experiment by Osakabe et al. (2017) used a sample series with strict mixing ratios, the effect of individual differences in DNA yield was not evaluated. Instead, we conducted a numerical experiment to verify the accuracy of the simultaneous parameter estimation under uncertainty in the individual DNA yield. The frequency of the R allele in the population, p, was set to 0.01, 0.05, 0.1, 0.25, 0.5 or 0.75.

For the sampling strategy, N bulk samples (the parameter “ntrap” in the R source code), each comprising n individuals (n was fixed among the samples: the parameter “npertrap” in the code), were generated by assuming random sampling from a wild population of a haploid organism. To assess how the estimation interval responds to the sample sizes, we evaluated the combination of N = {2, 4, 8, 16, 32, 64} and n = {4, 8, 16, 32, 64}, though the combinations with Nn>128 were excluded (Nn corresponds to “ntotal” in the code). The DNA quantities ($X_{R}$ and $X_{S}$) present in each bulk sample were generated as random numbers that followed the gamma distributions of Equation 3. To cover a plausible variability range of the DNA yield, the gamma shape parameter was varied as k = {1, 3, 9, 27}. Depending on the size of k, the gamma scale parameter was set at $\theta =1\times 10^{‐6}/k$ to fix the mean of the individual DNA yield to $1\times 10^{‐6}$. The termination threshold for qPCR, $X_{\Theta }$, was fixed at 1.

We fixed the other parameters due to the limitations of the computing resources. From the results of Experiment 1, $\delta_{T}$ = 1.2, $\delta_{B}$ = 0.24, z = 0.0016 and η = 0.97 were presupposed. As for the random errors in the PCR amplification process and/or the Cq measurement, $\sigma_{c}$ = 0.2 was assumed regardless of the initial template quantity. For each of the 624 parameter regions, the dummy data sets comprising *N* bulk samples were generated 1000 times independently with different random number seeds (i.e., 1000 replicates), for which the parameter estimation with freqpcr(…, beta = TRUE) of the freqpcr package version 0.3.1 was run on the R 3.6.1 environment. The simulation code is available in ESM 3.

For each parameter region, the success of the interval estimation was defined as the empirical probability that the freqpcr() function returned certain values other than NA (i.e., the diagonal of the Hessian matrix was not negative). There was no guarantee that the estimated confidence interval was accurate in each trial. The accuracy of the maximum‐likelihood estimate and the 95% confidence interval (i.e., the precision of the interval estimate) were assessed for each parameter region by pooling the estimates and the upper/lower limit values for the 1000 replicates to obtain the quantiles.

We also implemented the gamma distribution model as freqpcr(…, beta = FALSE). A numerical experiment with the gamma model was also conducted for the first 250 replicates, and the estimation accuracy was compared between the two assumptions. Furthermore, we also fitted the function with the settings freqpcr(…, *K* = 1), that is, assuming the gamma shape parameter was fixed at 1 (a.k.a. exponential distribution), in addition to the default simulation with all parameters (*p*, *k*, $\delta_{T}$ and $\sigma_{c}$) unknown. Further, the easiest way to estimate p derived from Equation 8 is to average the observed ΔΔCq values for *N* bulk samples and transform them as $\hat{p}={\left(1+\eta \right)}^{\left(‐\bar{\Delta \Delta \tau }\right)}$.

## 4 RESULTS

### 4.1 Estimation of auxiliary parameters and verification of homoscedasticity

Based on the Cq measures, the auxiliary parameters were estimated based on the RED‐ΔΔCq analysis of the I1017F mutation of *Tetranychus urticae*. As for the initial quantity of template DNA (the parameter “meanDNA” on the R code; defined as $X/X_{\Theta }$), the maximum‐likelihood estimate was 1.256 × 10^−6^ (95% confidence interval [CI]: 7.722 × 10^−7^ to 2.041 × 10^−6^). The relative quantity of the target gene to the housekeeping gene $\delta_{T}$ (targetScale) was estimated to be 1.170 (95% CI: 1.069–1.280). The locus‐independent change rate in the template quantity accompanying the restriction enzyme treatment $\delta_{B}$ (baseChange) was 0.2361 (95% CI: 0.2040–0.2731). The measurement error in the scale of Cq $\sigma_{c}$ (*SD*) was 0.2376 (95% CI: 0.2050–0.2755). The residue rate of the S allele after digestion *z* (zeroAmount) was 0.001564 (95% CI: 0.001197–0.002044). The efficiency of amplification per PCR cycle η (EPCR) was 0.9712 (95% CI: 0.9231–1.022).

**FIGURE 3:**
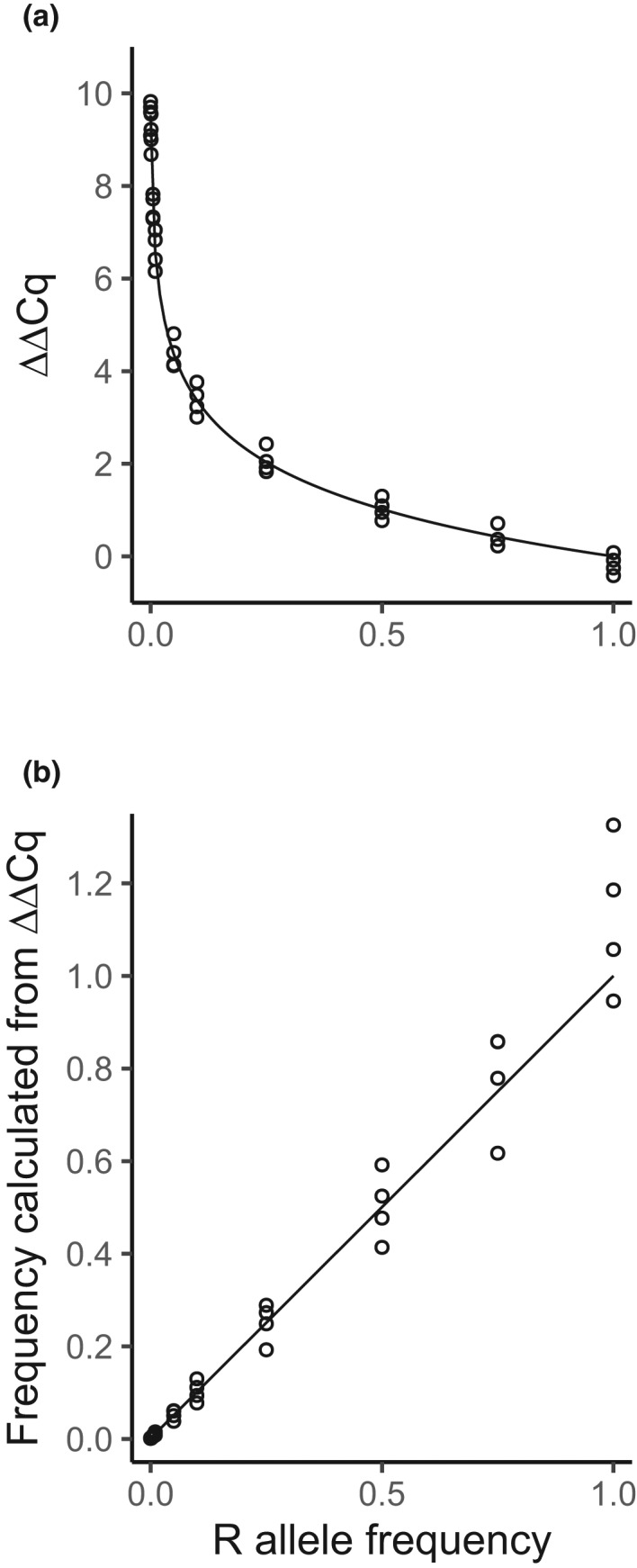
Relationship between the allele frequency in the sample and (a) the RED‐ΔΔCq measures and (b) the observed frequency calculated as (1 + *η*)^(−ΔΔCq)^, showing the results of etoxazole resistance in the two‐spotted spider mites. The lines are not the regression on the actual Cq measurement (shown as points), but the theoretical relationship between the true frequency of the R allele and the quantity defined as (a) $‐\text{ln}\left(z+Y_{R}\left(1‐z\right)\right)/\text{ln}\left(1+\eta \right)$ or (b) $z+Y_{R}\left(1‐z\right)$, where $Y_{R}=X_{R}/\left(X_{R}+X_{S}\right)$. Parameters are *z* = 0.00156 and *η* = 0.971

In the RED‐ΔΔCq analysis of the etoxazole resistance of *T*. *urticae*, the relationship between the true R allele frequency ($Y_{R}=X_{R}/\left(X_{R}+X_{S}\right)$ in the sample) and the corresponding Cq measures exhibited higher homoscedasticity in the scale of the measured ΔΔCq values rather than in (1 + *η*)^−ΔΔCq^, the transformation to ${\hat{Y}}_{R}$ (Figure 3). The linear regression of the ΔΔCq values on $‐\text{ln}\left[0.001564\times \left(1‐Y_{R}\right)+Y_{R}\right]/\text{ln}\left(1+0.971\right)$ showed high linearity (intercept = −0.07694, coefficient = 1.025, adjusted *R* ^2^ = 0.9936). The homoscedasticity of the coefficient of determination was not rejected at the 5% level of significance (Breusch–Pagan test: BP = 3.1577, *df* = 1, *p* = .07557) (Figure 3a). Conversely, the linear regression of 1.971^−ΔΔCq^ on $\left[0.001564\times \left(1‐Y_{R}\right)+Y_{R}\right]$ showed a slightly lower linearity (intercept = −0.008625, coefficient = 1.092, adjusted *R* ^2^ = 0.9709). The Breusch–Pagan test was highly significant (BP = 13.978, *df* = 1, *p* = .0001849), rejecting homoscedasticity (Figure 3b). These results suggest that it is easier to model the error structure of the RED‐ΔΔCq method on the scale of Cq values (logarithm) rather than frequency (linear scale).

### 4.2 Evaluation of the simultaneous estimation method with randomly generated data

For interval estimation of the population allele frequency, the estimation success probability was improved by fixing the size of the gamma shape parameter. Among the numerical simulations using freqpcr(…, beta = TRUE), conducted for 624 parameter regions with 1000 replicates, the 95% confidence intervals of *p* were returned in 70.6% and 94.5% when all parameters were unknown, and when the shape parameter was fixed as *k* = 1, respectively. The estimation success for the Cq measurement error, $\sigma_{c}$, was 69.6% and 97.6% in the beta distribution model with unknown *k* and *k* = 1, respectively. The relative quantity of the target gene, $\delta_{T}$, was 68.1% and 96.1%, respectively. The estimated success of *k* (when set unknown) was 59.9% with the beta distribution model, showing a lower performance than the other parameters. Conversely, the estimation of *p* is robust to the size of *k*, as we show later in this section.

**FIGURE 4:**
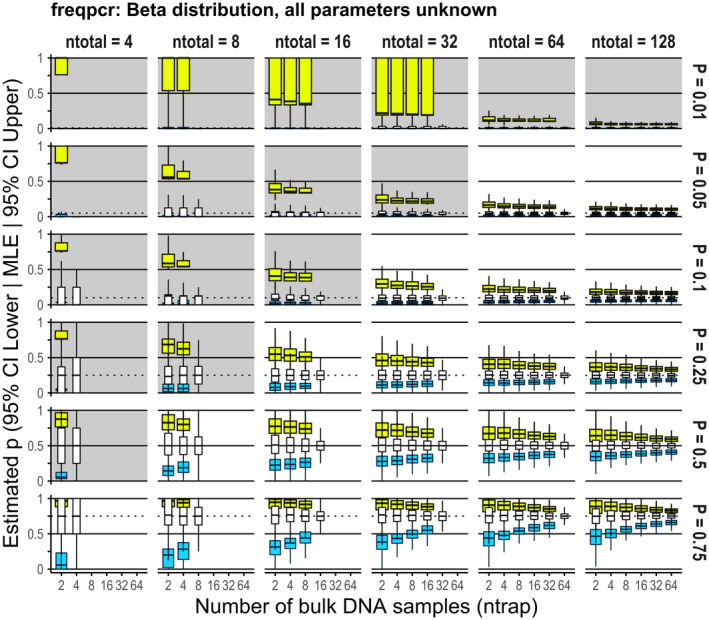
Estimation accuracy of the population allele frequency, *p*, with freqpcr() when the beta distribution was assumed, and all estimable parameters (P, K, targetScale and sdMeasure) were set as unknown. The result of numerical experiments is based on 1,000 dummy data sets per parameter region. The *x*‐axes correspond to *N*, or the “ntrap” parameter, the extent to which the collected individuals (ntotal) were divided into the bulk samples. The three box plots (white thin, blue and yellow wide) in each region show the maximum‐likelihood estimates (MLE), lower bound of the 95% confidence interval (CI) and the upper bound, respectively. In each boxplot, the horizontal line signifies the median of the simulations, hinges of the box show 25th and 75th percentiles, and the upper/lower whiskers correspond to the 1.5× interquartile ranges. The shaded facets show that the total sample sizes (ntotal) are smaller than 3/*p*

**FIGURE 5:**
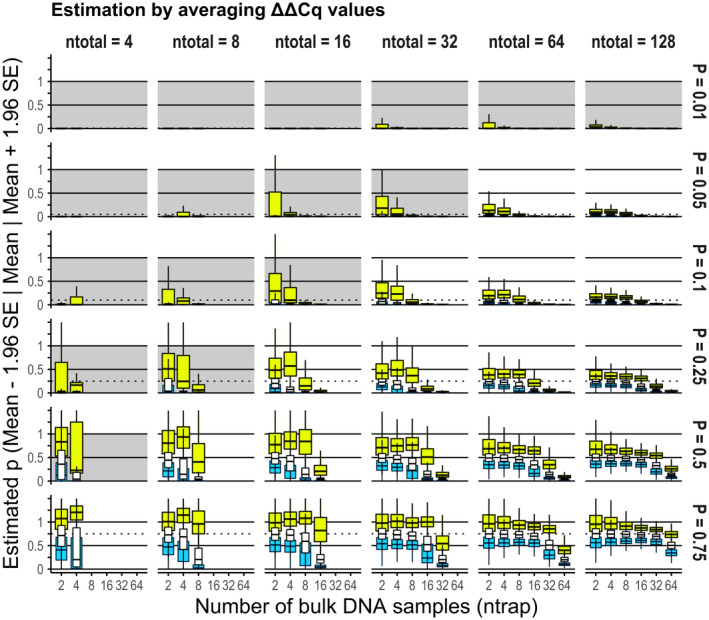
Estimation accuracy of the population allele frequency by simple averaging of ΔΔCq measures. The frequency was underestimated compared to its true value (horizontal broken line in each facet) as the samples were more divided. The three box plots (white thin, blue and yellow wide) in each region show the mean, lower bound of the 95% confidence interval (CI) and the upper bound, respectively. The Cq data set was derived from the numerical experiment of “beta distribution, all parameters unknown”

The estimation success of freqpcr()depended largely on the total sample size (*Nn* corresponding to the facet “ntotal” in the figures), as well as the level of *p* (Figures S1 and S2 for the beta and gamma models, with all parameters unknown). In each parameter region, the quantity $\text{Bin}\left(0|Nn,p\right)$ generally gives the probability that the whole sample contains no R individuals. When *Nn* is large enough, Nn>3/p is approximately the requirement for the total sample size to contain at least one R individual with 95% confidence, called the “rule of three” (Eypasch et al., 1995). The grey backgrounds in the facets of Figures 4 and 5 and S1–S7 signify the regions where the total sample sizes are smaller than the thresholds (e.g., 60 haploid individuals are required when *p* = .05). As shown in Figures S1 and S2, the parameter estimation often failed when *Nn* did not meet the rule of three. Once we exclude the parameter regions of Nn≤3/p, the estimation success rate of *p* with freqpcr(…, beta = TRUE) improved to 84.3% and 99.9% with all parameters unknown and assuming *k* = 1, respectively.

For the estimation accuracy of *p*, the freqpcr() function, which assumes a beta distribution, provides an unbiased estimator. Figure 4 and S3 show the estimated sizes of *p* using the beta model with all parameters unknown and assuming *k* = 1, respectively. Both settings demonstrated that the estimator converged to the true R frequency; the upper/lower bounds of the estimated 95% confidence intervals (yellow/blue boxes in each plot) became narrower as we increased the total sample sizes (*Nn*). According to the summary table (ESM 1: worksheet “Simulation_Result_Beta_all”), the 95% CI of *p* roughly falls within the range [*p*/3, 3*p*] when Nn>3/p. If the total sample size was doubled (*Nn* > 6/*p*), we obtained a narrower 95% CI between *p*/2 and 2*p*, which is considered satisfactory for practical interval estimation.

Although there was a larger contribution of increasing the total sample size (*Nn*), we obtained a narrower confidence interval of *p* as the samples were more divided under the given total sample size ($N/\left(\mathrm{Nn}\right)$ was large). However, if every individual was analysed separately, the interval estimation was only possible when *k* was fixed (see the regions of “sample division = ntotal” cases in Figure 4). In contrast, fixing the size of the gamma shape parameter to *k* = 1 scarcely affected the point estimates and intervals of *p*, as long as Nn>3/p is satisfied (Figure S3).

When we used the gamma distribution model (the number of replicates was 250), the interval estimation of *p* was also possible and unbiased (Figure S4). However, when we defined the point estimator of *p* as a simple average, that is, $\hat{p}={\left(1+\eta \right)}^{\left(‐\bar{\Delta \Delta \tau }\right)}$, it was strongly underestimated as the samples were more divided (Figure 5). The upper limit of the 95% CI often violated 1, suggesting that the “simple average of ΔΔCq” ± 1.96 SE is inadequate for the interval estimation based on the RED‐ΔΔCq method.

The calculation time and the number of iterations before convergence varied largely in the model settings and sample sizes (Figures S5–S7). Among the settings we tried, the beta model with fixed *k* was the fastest and converged within a few seconds in most parameter regions (median and 75th percentile: 0.32 and 0.69 s: Figure S6). It was three and >10 times faster than the beta (0.91 and 2.4 s: Figure S5) and gamma (3.0 and 15 s: Figure S7) model, respectively, with all parameters unknown. The calculation time generally increased as the data set size increased (larger *Nn*). It also increased as the sample was more divided in the beta distribution model because the marginal likelihood was calculated for each bulk sample (Figures S5 and S6). Conversely, the gamma distribution model (Figure S7) requires increased calculation time as the size of each bulk sample becomes larger (larger *n*_h_). This was considered because the combination of $\text{Bin}\left(m_{h}|n_{h},p\right)$ exploded when *n*_h_ was large.

Furthermore, the estimation accuracy of the shape parameter, *k*, was underestimated as the real size of the parameter increased (e.g., *k* = 27) when the gamma distribution model was applied (Figure S8B). Since the iterative fitting of the parameter in freqpcr() always starts internally from *k* = 1 (this was determined due to the calculation stability), this bias suggests that the likelihood function of $\psi_{G}$ (Equation 13) has little information on the size of *k* compared with *p*. Thereafter, *k* tends to stay at its initial value, suggesting that the gamma model is less suitable for the simultaneous estimation of *p* and *k*. Unlike the gamma version, the fitting of *k* with freqpcr(beta = TRUE) was satisfactory when we divided the total samples into more bulk samples. However, the initial value dependence was still observed, especially when *p* or *N* was small (Figure S8A), which may be because the estimation of *k* via $\text{Beta}\left(m_{h}k,\left(n_{h}‐m_{h}\right)k\right)$ in Equation 14 is comparable with measuring the overdispersion of $Y_{h,R|m_{h}}$, which is only possible when multiple bulk samples contain both R and S alleles.

## 5 DISCUSSION

In the present study, we developed a statistical model to estimate the population allele frequency based on qPCR across multiple bulk samples to address the issues facing the conventional point estimator for allele frequency which averages the observed ΔΔCq values $\hat{p}={\left(1+\eta \right)}^{\left(‐\bar{\Delta \Delta \tau }\right)}$. This conventional method sometimes exceeds 1 when the frequency of the target allele is close to 1. Furthermore, when quantifying the rare mutant allele in a population, most bulk samples contain only the wild‐type allele. The conventional $\hat{p}$ is vulnerable to many zero samples, which makes the frequency estimation more difficult when *p* is small. To circumvent these problems, our interval estimation explicitly models the number of individuals contained in each bulk sample (the binomial assumption) as well as the individual DNA yields (the gamma assumption), thereby obtaining the interval estimate over the entire range 0<p<1.

The explicit modelling of individuals also allows sample division to various degrees, which helps us balance our sampling strategy on the cost–precision tradeoff. We can achieve higher precision (narrower confidence interval) by increasing the total sample size, $\sum_{h=1}^{N}n_{h}$, although it also increases the costs of sample collection and laboratory work, including library preparation and PCR analysis. Although it is possible to extract DNA from dead bodies obtained via mass trapping, a larger sample size still imposes a higher handling cost if we analyse the collected organisms individually via nonquantitative PCR (Toda et al., 2017; Uesugi et al., 2016).

The combination of mass trapping and bulk qPCR analysis offers a solution by collecting more individuals and pooling them, resulting in higher precision with less work than individual PCR. For instance, we sampled 16 individuals from the population with an allele frequency of *p* = .05 and analysed two individuals once in the numerical experiment (Figure 4: facet of ntotal = 16, sample division = 8). The lower and upper limits of the 95% CI *p* were estimated to be .0087 and .34, respectively, using freqpcr(…, beta = TRUE) (as the medians of the 1000 independent trials). We also simulated the case of ntotal = 64 and sample division = 4 (i.e., analysed 16 individuals together) and found the upper and lower limits to be 0.015 and 0.15, respectively. Thus, we improved the precision of the interval estimate with half the handling effort.

In nonquantitative PCR, sample pooling is considered as a tool for the detection of rare (c)DNA in the population with practical labour requirements and has been used as a high‐throughput prescreening system for many samples, such as in clinical examinations (Taylor et al., 2010; Yelin et al., 2020). In some fields, such as plant quarantine, frequency estimation is not realistic as the assumed frequency range is low (p≤.001). According to the “rule of three,” the required sample size is 3,000 to contain at least one product contaminated with pests or unapproved genetically modified seeds when *p* = .001. In the inspection routine of plant quarantine, group testing based on nonquantitative PCR is designed to ensure the contamination is not detected at a certain consumer risk (Yamamura et al., 2019). Yamamura and Hino (2007) proposed a semiquantitative method to estimate the upper limit of the population allele frequency based on the proportion of bulk samples detected as “positive.”

Overall, there has been a gap in methodology between the frequency estimation based on the individual PCR and the non‐ or semiquantitative PCR based on the nonquantitative bulk PCR. Although individual PCR provides the highest estimation precision following binomial distribution, it is only available at a higher *p*; it becomes labour‐intensive once we try to quantify rare alleles. The nonquantitative bulk PCR can be applied to a lower range of *p*, but the precision is generally low. Bridging the gap, ΔΔCq‐based qPCR analyses for multiple bulk samples offer an allele frequency estimation in the mid‐ to low range (*p* = .01 to .25), which is considered a critical range for decision‐making in some fields like pesticide resistance management (Sudo et al., 2018; Takahashi et al., 2017).

Although this study exemplified resistance genes, the likelihood model of Equation 11 can also be applied for other qPCR protocols; the prerequisite is that the point estimate of the sample allele frequency is obtained in the form of the ΔΔCq measure. If both the nonspecific and specific primer sets are available to amplify the “wild type + mutant” and “mutant” alleles at the target locus, they can replace the control (undigested) and test (digested) samples, which are equivalent to *X* ^TW^ in Equation 5 and *X* ^TD^ in Equation 6, respectively. However, there is a caveat in determining which allele should be amplified with a specific primer set and which affects the estimation accuracy due to the intrinsic nature of (1 + *η*)^−ΔΔ^ *^τ^*. As shown, the 95% CIs were broader when *p* = .75 than when *p* = .25 (Figure 4), and the precision was not symmetric around 0.5, but more precise when the frequency was low; that is, one should design a specific primer set to amplify the allele that would be rare in the population to improve the signal‐to‐noise ratio.

The maximum‐likelihood estimation with freqpcr() relies on the assumption that the quantities of the S and R alleles in each bulk sample independently follow a gamma distribution and that their quotient is expressed using a beta distribution. Although the freqpcr() function with the gamma and beta distributions both showed an unbiased estimation of *p*, the beta model was advantageous regarding calculation time and the number of iterations before convergence. Fixing the size of the gamma shape parameter *k* further accelerated the optimization, owing to the robustness of *p* to the size of *k*. However, once the size of *k* was fixed much larger than the actual size of the gamma shape parameter (i.e., the individual DNA yield was regarded as almost a fixed value), the iterative optimization using the nlm() function sometimes returned an error. Therefore, one should start with a smaller shape parameter, for example *k* = 1 (the exponential distribution: Figure S3), which is currently the default setting of the freqpcr package.

In qPCR applications for diagnostic use, ΔΔCq is often used with calibration. One popular method involves technical replicates; each sample is dispensed and analysed using qPCR multiple times, which negates the Cq measurement error. The measurement error obeys a homoscedastic normal distribution in the Cq scale, as shown in Experiment 1. Thus, a simple solution is to average the Cq values measured for each bulk sample before the estimation with freqpcr(), although the estimated size of $\sigma_{c}$ changes from its original definition in Equation 7. However, it is trivial if the number of technical replicates is unified between bulk samples. Besides, the comparison of Cq values is sometimes conducted on more than one internal reference as there is no guarantee that the expression level of a “housekeeping gene” is always constant (Vandesompele et al., 2002). Future updates of freqpcr() will handle multiple internal references.

Recent development in next‐generation sequencing (NGS) enables us to conduct individual‐based analysis on hundreds of samples in a single run (sample multiplexing) (Quail et al., 2012). Although high‐throughput genotyping might replace the PCR‐based allele‐frequency estimation in the future, it has not yet become fully available for many practitioners, especially of agricultural, environmental and public health sectors of local governments as well as small businesses. As genotyping with NGS is often performed in a large lot due to cost considerations, it may not be suitable when the user needs to know the results in short time intervals, such as in plant quarantine and regional pesticide resistance monitoring (Sonoda et al., 2017; Yamamura & Hino, 2007). As long as qPCR is used to estimate population allele frequency, the use of statistical inferences on the bulk samples, as presented in this study, will continue to be a realistic option for regional/temporal allele monitoring. Likewise, our model approach to the individual DNA yields can also be extended to the NGS‐based estimation procedures since the gamma distribution has been used to quantify environmental and forensic DNA (Cowell et al., 2007; Fukaya et al., 2021).

## AUTHOR CONTRIBUTIONS

M.S. designed the study, made the statistical models and R package, and analysed the data. M.O. conducted the laboratory work. Both authors drafted the final version of the manuscript.

## CONFLICT OF INTEREST

The authors declare no conflicts of interest associated with this manuscript.

## Supporting information

Appendix S1

Appendix S2

Appendix S3

Appendix S4

## Data Availability

The R package source is available at https://github.com/sudoms/freqpcr. The mite data set from Osakabe et al. (2017) (10.6084/m9.figshare.16870816.v1) and the output data of the numerical experiment (10.6084/m9.figshare.c.5258027.v1) are available at fig‐share.com. The source code for the figures, including the mite data set, is available as Appendix S1 and electronic supplementary materials [Link], [Link], [Link]. Appendix S1: Formularization in the case of diploidy, including supplementary figures S1‐S8. ESM 1 (Appendix S2): RED‐ΔΔCq data set from Osakabe et al. (2017). The last two worksheets show the results of the numerical simulation on the required sample size for the interval estimation using freqpcr(…, beta = TRUE). ESM 2 (Appendix S3): R source code for Experiment 1 (Figure 3), including a brief guide to the “freqpcr” package. ESM 3 (Appendix S4): R source code for the numerical simulation (Experiment 2) and the codes for Figure 4 onwards.
