# Supplementary material for: freqpcr: Estimation of population allele frequency using qPCR ΔΔCq measures from bulk samples
Source: Mol Ecol Resour. 2021 Dec 9;22(4):1380–93. doi: 10.1111/1755-0998.13554 (PMC9300209; doi:10.1111/1755-0998.13554)
Supplement: Supplementary file 1 — Appendix S1 [file MEN-22-1380-s001.docx]

Supplemental Information for:
freqpcr: estimation of population allele frequency using qPCR ΔΔCq measures from bulk samples

Masaaki Sudo, Masahiro Osakabe

Table of Contents

[Appendix 2](#_Toc86178400)

[Appendix S1: Case of Diploidy 2](#_Toc86178401)

[Parameter estimation 2](#_Toc86178402)

[Supplementary figures 4](#_Toc86178403)

[Figure S1 Probability of estimation success with freqpcr() 4](#_Toc86178404)

[Figure S2 Probability of estimation success with freqpcr() 5](#_Toc86178405)

[Figure S3 Estimation accuracy of *p*, the population allele frequency (beta distribution model, *K* = 1) 6](#_Toc86178406)

[Figure S4 Estimation accuracy of *p* (gamma distribution model) 6](#_Toc86178407)

[Figure S5 Calculation time and number of iterations (beta distribution model) 7](#_Toc86178408)

[Figure S6 Calculation time and number of iterations (beta distribution model, *K* = 1) 8](#_Toc86178409)

[Figure S7 Calculation time and number of iterations (gamma distribution model) 9](#_Toc86178410)

[Figure S8 Estimation accuracy of the gamma shape parameter 10](#_Toc86178411)

These are distributed in separate files.

ESM 1

RED-ΔΔCq dataset from Osakabe et al. (2017). The last two worksheets show the results of the numerical simulation on the required sample size for the interval estimation using freqpcr(..., beta = TRUE).

ESM 2

R source code for Experiment 1 (Figure 3), including a brief guide to the “freqpcr” package.

ESM 3

R source code for the numerical simulation (Experiment 2) and the codes for Figures 4 and after.

The R package source is available at: https://github.com/sudoms/freqpcr

Latest package source at submission (v0.3.5): https://doi.org/10.6084/m9.figshare.16828912.v1

The mite dataset from Osakabe et al. (2017): https://doi.org/10.6084/m9.figshare.16870816.v1

The output data of the numerical experiment: https://doi.org/10.6084/m9.figshare.c.5258027.v1

# Appendix

## Appendix S1: Case of Diploidy

Although we considered sampling from haploid organisms, many insects and vertebrates are diploid. Let us consider that the population of a diploid insect species has the R allele frequency $p$, from which we collected *n* individuals. The bulk sample then consists of $m_{1}$ ($m_{1}=0,1,,...,n$) individuals of RR homozygotes, $n-m_{1}-m_{0}$ RS heterozygotes, and $m_{0}$ ($m_{0}=0,1,,...,n$) SS homozygotes $\left( m_{1}+m_{0}\leq n \right)$. The joint probability of obtaining $\left\{ m_{1},m_{0} \right\}$ obeys the trinomial distribution with probabilities $p^{2}$ and $\left( 1-p \right)^{2}$

$$\text{Tri}\left( m_{1},m_{0} | n,p^{2},\left( 1-p \right)^{2} \right)=\frac{n!}{m_{1}!m_{0}!\left( n-m_{1}-m_{0} \right)!}\cdot p^{2m_{1}}\cdot\left( 1-p \right)^{2m_{0}}\cdot\left( 2p-2p^{2} \right)^{\left( n-m_{1}-m_{0} \right)}.$$

Eq. 15

The total R allele in the bulk sample comes from two R/R sets contained in the $m_{1}$ homozygotes and a single set of R from the $n-m_{1}-m_{0}$ heterozygotes. Likewise, two S/S sets from $m_{0}$ homozygotes and a single S set from the $n-m_{1}-m_{0}$ heterozygotes constitute the total S body. Note that the yields of R and S from these heterozygotes would be the same unless there is a genotype-dependent systematic error in the extraction efficiency.

Let us define the amount of DNA copies per genome: the random variable $X_{*\in\left( \text{S},\text{R} \right)|\text{homo}}$ for the yield of S or R from the homozygotes, and $X_{*\in\left( \text{S},\text{R} \right)|\text{hetero}}$ for S or R from the heterozygotes. As in the case of haploidy, $X_{\text{R}}$ and $X_{\text{S}}$ denote the allele contents in the bulk sample; they are the linear combinations of $X_{*|\text{homo}}$ and $X_{*|\text{hetero}}$:

$$\begin{matrix} X_{\text{R}}=2\times X_{\text{R|homo}}+X_{\text{R|hetero}}, & X_{\text{S}}=X_{\text{S|hetero}}+2\times X_{\text{S|homo}}, \end{matrix}$$

$$\begin{matrix} 2\times X_{\text{R|homo}}\sim\text{Ga}\left( m_{1}k,2\theta\right), & X_{\text{R|hetero}}\sim\text{Ga}\left( \left( n-m_{1}-m_{0} \right)k,\theta\right), \\ X_{\text{S|hetero}}=X_{\text{R|hetero}}, & 2\times X_{\text{S|homo}}\sim\text{Ga}\left( m_{0}k,2\theta\right). \end{matrix}$$

Eq. 16

### Parameter estimation

There are$n-i+1$ cases from $m_{0}=0$ to $m_{0}=n-i$ when the number of RR homozygotes is given by $m_{1}=i$. The segregation ratio in the bulk sample has $\sum_{i=0}^{n} \left( n-i+1 \right)$ total combinations. For each combination of $n$, $m_{0}$, and $m_{1}$, Eq. 16 gives the probability of obtaining the $\Delta$Cq measures in Eq. 11. However, a drawback arises from the constraint of the amounts of R and S possessed by heterozygotes. The applicability of the likelihood model (Eq. 13 or Eq. 14 in the main text) depends largely on the independence of $X_{\text{R}}$ and $X_{\text{S}}$. If we define the likelihood using Eq. 16 as it was, we must convolve the DNA amounts not on the two-dimensional parameter space spanned by $X_{\text{R}}$ and $X_{\text{S}}$, but a three-dimensional space by $X_{\text{R|homo}}$, $X_{\text{S|hetero}}=X_{\text{R|hetero}}$, and $X_{\text{S|homo}}$, which would increase the calculation time.

Therefore, we removed the constraint and assumed that $X_{\text{R|}\text{*}}$ and $X_{\text{S|}\text{*}}$ were distributed independently and identically; that is, instead of the heterozygotes, we captured $n-m_{1}-m_{0}$ individuals of haploid R and another $n-m_{1}-m_{0}$ individuals of haploid S separately. Regarding homozygotes, we also assumed that we captured $2m_{1}$ R haploids and $2m_{0}$ S haploids instead of $m_{1}$ RR and $m_{0}$ SS, respectively. Then,

$$\begin{matrix} X_{\text{R|homo}}\sim\text{Ga}\left( 2m_{1}k,\theta\right), & X_{\text{R|hetero}}\sim\text{Ga}\left( \left( n-m_{1}-m_{0} \right)k,\theta\right), \\ X_{\text{S|hetero}}\sim\text{Ga}\left( \left( n-m_{1}-m_{0} \right)k,\theta\right) i.i.d., & X_{\text{S|homo}}\sim\text{Ga}\left( 2m_{0}k,\theta\right). \end{matrix}$$

Eq. 17

Finally, we can approximate the DNA amounts of a diploid organism in the bulk sample by simply substituting Eq. 3 in the main text:

$$\begin{matrix} X_{\text{R}}\sim\text{Ga}\left( \left( n+m_{1}-m_{0} \right)k,\theta\right), & X_{\text{S}}\sim\text{Ga}\left( \left( n-m_{1}+m_{0} \right)k,\theta\right). \end{matrix}$$

Eq. 18

In addition, at probability $\text{Bi}\text{n}\left( 0|{2n}_{h},p \right)$, all (hypothetically haploid) individuals become S or R; in that case, there is no need to convolve the DNA amounts.

# Supplementary figures

## Figure S1 Probability of estimation success with freqpcr()

Figure S1 Probability of estimation success with freqpcr(). The beta distribution was assumed, and all estimable parameters (P, K, targetScale, and sdMeasure) were set as unknown. The shaded boxes in the background show the frequency ranges where the total sample sizes (ntotal) are smaller than 3/*p*.

## Figure S2 Probability of estimation success with freqpcr()

Figure S2 Probability of estimation success with freqpcr(). The gamma distributions were assumed, and all estimable parameters were set as unknown. The function often failed to calculate the CIs for *k* when npertrap (individuals in each bulk sample) were larger.

## Figure S3 Estimation accuracy of *p*, the population allele frequency (beta distribution model, *K* = 1)

Figure S3 Estimation accuracy of the population allele frequency, *p*, with freqpcr() when the beta distribution was assumed, considering K = 1.

## Figure S4 Estimation accuracy of *p* (gamma distribution model)

Figure S4 Estimation accuracy of *p* with freqpcr() when gamma distributions were assumed and all estimable parameters were set as unknown.

## Figure S5 Calculation time and number of iterations (beta distribution model)

Figure S5 Calculation time (A) and number of iterations (B) until the freqpcr() function converges. The beta distribution was assumed, and all estimable parameters were set as unknown.

## Figure S6 Calculation time and number of iterations (beta distribution model, *K* = 1)

Figure S6 Calculation time (A) and number of iterations (B) until the freqpcr() function converges. The beta distribution was assumed, fixing the gamma shape parameter K = 1.

## Figure S7 Calculation time and number of iterations (gamma distribution model)

Figure S7 Calculation time (A) and number of iterations (B) until the freqpcr() function converges, assuming gamma distributions. All estimable parameters were set as unknown.

## Figure S8 Estimation accuracy of the gamma shape parameter

Figure S8 Estimation accuracy of *k* (the gamma shape parameter) in the simulation, showing the maximum likelihood estimate by freqpcr() divided by the actual parameter size.
