# Supplementary material for: freqpcr: Estimation of population allele frequency using qPCR ΔΔCq measures from bulk samples
Source: Mol Ecol Resour. 2021 Dec 9;22(4):1380–93. doi: 10.1111/1755-0998.13554 (PMC9300209; doi:10.1111/1755-0998.13554)
Supplement: Supplementary file 3 — Appendix S3 [file MEN-22-1380-s002.pdf]

freqpcr: estimation of population allele frequency using qPCR ΔΔCq measures from bulk samples

Masaaki Sudo, Masahiro Osakabe

ORCID ID:

- 0000-0001-9834-9857 (Masaaki Sudo)
- 0000-0002-2246-3431 (Masahiro Osakabe)

```
R package name: freqpcr
Short description: interval estimation of population allele frequency based
on ΔΔCq measures of quantitative PCR over bulk samples
Package author & maintainer: Masaaki Sudo (NARO, JAPAN)
https://github.com/sudoms/freqpcr
```

ESM 2

R source code for Experiment 1 (Figure 3), including a brief guide to the "freqpcr" package.

```
Tested environment:

> sessionInfo()
R version 3.6.1 (2019-07-05)
Platform: x86_64-w64-mingw32/x64 (64-bit)
Running under: Windows 10 x64 (build 18363)

Matrix products: default

Random number generation:
 RNG:     Mersenne-Twister
 Normal:  Inversion
 Sample:  Rounding

locale:
[1] LC_COLLATE=Japanese_Japan.932  LC_CTYPE=Japanese_Japan.932
```

```
LC_MONETARY=Japanese_Japan.932 LC_NUMERIC=C
[5] LC_TIME=Japanese_Japan.932

attached base packages:
[1] parallel  stats     graphics  grDevices utils     datasets  methods
base

other attached packages:
 [1] installr_0.22.0 furrr_0.1.0     future_1.16.0   forcats_0.4.0
stringr_1.4.0   dplyr_0.8.4     purrr_0.3.3
 [8] readr_1.3.1     tidyr_1.0.2     tibble_2.1.3    ggplot2_3.2.1
tidyverse_1.3.0

loaded via a namespace (and not attached):
 [1] tidyselect_1.0.0 listenv_0.8.0    haven_2.2.0      lattice_0.20-40
colorspace_1.4-1 vctrs_0.2.3      generics_0.0.2
 [8] utf8_1.1.4       rlang_0.4.5      pillar_1.4.3     glue_1.3.1
withr_2.1.2      DBI_1.1.0        dbplyr_1.4.2
[15] modelr_0.1.6     readxl_1.3.1     lifecycle_0.1.0  munsell_0.5.0
gtable_0.3.0     cellranger_1.1.0 rvest_0.3.5
[22] codetools_0.2-16 fansi_0.4.1      broom_0.5.4      Rcpp_1.0.3
scales_1.1.0     backports_1.1.5  jsonlite_1.6.1
[29] fs_1.3.1         hms_0.5.3        digest_0.6.25    stringi_1.4.6
grid_3.6.1       cli_2.0.1        tools_3.6.1
[36] magrittr_1.5     lazyeval_0.2.2   crayon_1.3.4     pkgconfig_2.0.3
xml2_1.2.2       reprex_0.3.0     lubridate_1.7.4
[43] assertthat_0.2.1 httr_1.4.1       rstudioapi_0.11  R6_2.4.1
globals_0.12.5   nlme_3.1-144     compiler_3.6.1
```

`freqpcr()` package installation

```
# for the first time
library(devtools)
Sys.setenv(R_REMOTES_NO_ERRORS_FROM_WARNINGS="true") # If you had an error
(converted from warning)
install_github("sudoms/freqpcr")

# or install by hand
install.packages("freqpcr_0.3.5.tar.gz", repos=NULL, type="source")

# load the package
library(freqpcr); packageVersion("freqpcr");
```

Part I Procedure to determine parameters

Experiment 1

Example dataset: DNA solution with known mixing ratio

```
Copyright by Masahiro Osakabe (Kyoto University) 2017

Osakabe, M., T. Imamura, R. Nakano, S. Kamikawa, M. Tadatsu, Y. Kunimoto,
and M. Doi. 2017.
Combination of restriction endonuclease digestion with the DeltaDeltaCt
method in real-time PCR to monitor etoxazole resistance allele frequency in
the two-spotted spider mite.
Pesticide biochemistry and physiology 139:1-8.
```

DNA was extracted from the etoxazole resistant (R) or susceptible (S) populations of the two-spotted spider mite, *Tetranychus urticae*.

```
# reading raw experiment data
library(readxl)
library(tidyverse)
mite.raw <- readxl::read_xlsx(  path="ESM1_mite.xlsx", sheet="Rawdata",
                                col_types=c(rep("text", 1), rep("numeric",
8))  )
data.frame(mite.raw)
```

```
> data.frame(mite.raw)
    Gene numR numS Digest FreqTrue     Cq.1     Cq.2     Cq.3     Cq.4
1   CHS1   50    0      1    1.000 21.76011 22.46276 21.67745 21.89234
2   CHS1   50   50      1    0.750 22.22055 22.73058 22.49938 22.13801
3   CHS1   50   50      1    0.500 22.55539 22.92551 23.13384 22.67460
4   CHS1   50   50      1    0.250 23.72164 24.22364 24.21292 23.96121
5   CHS1   50   50      1    0.100 25.02524 25.64115 25.37562 24.97376
6   CHS1   50   50      1    0.050 26.22723 26.48074 26.38646 25.98100
7   CHS1   50   50      1    0.010 28.86382 28.44911 28.68426 28.17910
8   CHS1   50   50      1    0.005 29.23326 29.45677 29.40738 28.98874
9   CHS1   50   50      1    0.001 30.36377 31.16006 30.66308 30.84527
10  CHS1    0   50      1    0.000 30.91856 31.39115 31.86532 31.48347
11  CHS1   50    0      0    1.000 19.76440 19.93363 19.81920 19.58301
```

```
  12 GAPDH   50    0      1    1.000 22.25536 22.78204 22.21400 22.25800
  13 GAPDH   50   50      1    0.750 22.09678 22.42058 22.39418 22.19650
  14 GAPDH   50   50      1    0.500 21.70864 22.02676 22.48203 22.00767
  15 GAPDH   50   50      1    0.250 21.91582 22.19761 22.41978 22.41461
  16 GAPDH   50   50      1    0.100 21.78486 22.27926 22.26315 22.25005
  17 GAPDH   50   50      1    0.050 22.06956 22.07313 22.36781 22.14638
  18 GAPDH   50   50      1    0.010 22.27531 21.80223 22.39260 22.30971
  19 GAPDH   50   50      1    0.005 21.75721 22.03858 22.23731 21.94482
  20 GAPDH   50   50      1    0.001 21.92781 22.00806 21.78034 21.91004
  21 GAPDH    0   50      1    0.000 22.07731 22.09051 22.16355 22.17278
  22 GAPDH   50    0      0    1.000 20.00866 20.33493 19.94018 19.86679
```

```
Digest: The DNA sample was digested with Taq1 endonuclease before qPCR.
FreqTrue: exact frequency of the R (acaricide resistant) alleles.
Cq. 1 ~ 4 are the replicates.
```

data format 1: "unpaired"

Desired data unit used in the RED-ΔΔCq analysis is a 'quartet', the 2x2 combination of (target- or housekeeping-gene) x (undigested or digested). A quartet is needed to calculate a conventional ΔΔCq value. If all replicates of each experimental setting are fulfilled with the complete observations of the 2x2 combination, the dataset is easily treated in the "paired" format (see below).

An "unpaired" format is used when some of the experimental settings (mixing ratio, replicates, etc.) lacks the complete combination of the 2x2 condition. For instance, the dataset from Osakabe et al. (2017) had 10 mixing ratios, `R/(S+R) = c(0, 1, 0.001, 0.005, 0.01, 0.05, 0.1, 0.25, 0.5, 0.75)`, and four replicates. However, qPCR on the undigested samples were only conducted with mixing ratio=1.

The dataset is thus incomplete (i.e., the undigesed data must be recycled to calculate the conventional ΔΔCq values), though it is often the case in real operations. Such kind of a dataset is often prepared as a "longer" formatted tidy data as described here.

We can also prepare it as a "wider" tidy data allowing missing values, which is described in the next "paired" format.

```
# "mite.long" is an intermediate data format.
mite.long <- mite.raw %>%
    tidyr::pivot_longer(cols=starts_with("Cq"), names_to=c("Type", "Repl"),
                        values_to=c("Cq"), names_sep="\\.",
```

```
names_ptypes=list(Repl=factor()))
mite.long

mite.unpaired <- mite.long %>%
    dplyr::mutate(Gene=as.numeric(factor(Gene, levels=c("GAPDH", "CHS1")))
- 1) %>%
    dplyr::arrange(desc(FreqTrue), Repl, Gene, Digest) %>%
    dplyr::select(FreqTrue, Repl, Gene, Digest, Cq)
mite.unpaired

readr::write_csv(mite.unpaired, "mite.unpaired.csv")
```

The following `mite.unpaired` shows the shape of a "longer" tidy data.

```
> mite.long
# A tibble: 88 x 8
   Gene   numR  numS Digest FreqTrue Type  Repl     Cq
   <chr> <dbl> <dbl>  <dbl>    <dbl> <chr> <fct> <dbl>
 1 CHS1     50     0      1     1    Cq    1      21.8
 2 CHS1     50     0      1     1    Cq    2      22.5
 3 CHS1     50     0      1     1    Cq    3      21.7
 4 CHS1     50     0      1     1    Cq    4      21.9
 5 CHS1     50    50      1     0.75 Cq    1      22.2
 6 CHS1     50    50      1     0.75 Cq    2      22.7
 7 CHS1     50    50      1     0.75 Cq    3      22.5
 8 CHS1     50    50      1     0.75 Cq    4      22.1
 9 CHS1     50    50      1     0.5  Cq    1      22.6
10 CHS1     50    50      1     0.5  Cq    2      22.9
# ... with 78 more rows

> mite.unpaired
# A tibble: 88 x 5
   FreqTrue Repl   Gene Digest    Cq
      <dbl> <fct> <dbl>  <dbl> <dbl>
 1        1 1         0      0  20.0
 2        1 1         0      1  22.3
 3        1 1         1      0  19.8
 4        1 1         1      1  21.8
 5        1 2         0      0  20.3
 6        1 2         0      1  22.8
 7        1 2         1      0  19.9
 8        1 2         1      1  22.5
 9        1 3         0      0  19.9
10        1 3         0      1  22.2
# ... with 78 more rows
```

data format 2: "paired," allowing missing values

The data format called "paired" here is a kind of "wider" tidy data. The combinations of (target- or housekeeping-gene) x (undigested or digested) are provided as separated data columns.

There are two types of "paired" Cq data format. One is allowing missing values, where unobserved Cq data are filled as NA (not available).

```
mite.paired <- mite.long %>%
    tidyr::pivot_wider( names_from=c("Type", "Digest", "Gene"),
names_sep=".",
                        values_from="Cq" ) %>%
    dplyr::select(FreqTrue, Repl, target0=Cq.0.CHS1, target1=Cq.1.CHS1,
housek0=Cq.0.GAPDH, housek1=Cq.1.GAPDH) %>%
    dplyr::mutate(del.w=target0-housek0, del.d=target1-housek1) %>%
    dplyr::mutate(deldel=del.d-del.w) %>%
    dplyr::mutate(FreqCalc=2^-deldel)
mite.paired

readr::write_csv(mite.paired, "mite.paired.csv")
```

```
> mite.paired
# A tibble: 40 x 10
   FreqTrue Repl  target0 target1 housek0 housek1  del.w   del.d  deldel
FreqCalc
      <dbl> <fct>   <dbl>   <dbl>   <dbl>   <dbl>  <dbl>   <dbl>   <dbl>
<dbl>
 1     1    1        19.8    21.8    20.0    22.3 -0.244 -0.495  -0.251
1.19
 2     1    2        19.9    22.5    20.3    22.8 -0.401 -0.319   0.0820
0.945
 3     1    3        19.8    21.7    19.9    22.2 -0.121 -0.537  -0.416
1.33
 4     1    4        19.6    21.9    19.9    22.3 -0.284 -0.366  -0.0819
1.06
 5     0.75 1        NA      22.2    NA      22.1 NA      0.124  NA
NA
 6     0.75 2        NA      22.7    NA      22.4 NA      0.310  NA
NA
 7     0.75 3        NA      22.5    NA      22.4 NA      0.105  NA
```

```
NA
 8      0.75 4          NA       22.1     NA       22.2 NA      -0.0585 NA
NA
 9      0.5  1          NA       22.6     NA       21.7 NA       0.847  NA
NA
10      0.5  2          NA       22.9     NA       22.0 NA       0.899  NA
NA
# ... with 30 more rows
```

data format 2': "paired and recycled"

If we adhere to calculate the conventional ΔΔCq indices, the missing Cq values need to be filled with ones of the correspinding experimental settings (data recycling).

As for the mite dataset of Osakabe et al. (2017), those unobserved Cq data were copied from the corresponding observations when `FreqTrue == 1`.

```
# "mite.paired.middle" is the intermediate data generated from "mite.long"
mite.paired.middle <- mite.long %>%
    dplyr::group_by(Gene, Repl) %>%
    tidyr::pivot_wider( names_from=c("Type", "Digest"), names_sep=".",
values_from="Cq" ) %>%
    dplyr::mutate(Cq.0=mean(Cq.0, na.rm=TRUE))
mite.paired.middle

mite.paired.recycled <- mite.paired.middle %>%
    tidyr::pivot_wider( names_from=c("Gene"), names_sep=".", values_from=c
("Cq.1", "Cq.0") ) %>%
    dplyr::select(FreqTrue, Repl, target0=Cq.0.CHS1, target1=Cq.1.CHS1,
housek0=Cq.0.GAPDH, housek1=Cq.1.GAPDH) %>%
    dplyr::mutate(del.w=target0-housek0, del.d=target1-housek1) %>%
    dplyr::mutate(deldel=del.d-del.w) %>%
    dplyr::mutate(FreqCalc=2^-deldel)
mite.paired.recycled

readr::write_csv(mite.paired.recycled, "mite.paired.recycled.csv")
```

```
> mite.paired.middle
# A tibble: 80 x 7
# Groups:   Gene, Repl [8]
   Gene   numR  numS FreqTrue Repl   Cq.1  Cq.0
   <chr> <dbl> <dbl>    <dbl> <fct> <dbl> <dbl>
```

```
 1 CHS1     50     0     1     1       21.8  19.8
 2 CHS1     50     0     1     2       22.5  19.9
 3 CHS1     50     0     1     3       21.7  19.8
 4 CHS1     50     0     1     4       21.9  19.6
 5 CHS1     50    50     0.75  1       22.2  19.8
 6 CHS1     50    50     0.75  2       22.7  19.9
 7 CHS1     50    50     0.75  3       22.5  19.8
 8 CHS1     50    50     0.75  4       22.1  19.6
 9 CHS1     50    50     0.5   1       22.6  19.8
10 CHS1     50    50     0.5   2       22.9  19.9
# ... with 70 more rows

> mite.paired.recycled
# A tibble: 40 x 10
# Groups:   Repl [4]
   FreqTrue Repl  target0 target1 housek0 housek1  del.w   del.d   deldel
FreqCalc
      <dbl> <fct>   <dbl>   <dbl>   <dbl>   <dbl>  <dbl>   <dbl>   <dbl>
<dbl>
 1      1    1        19.8    21.8    20.0    22.3 -0.244 -0.495  -0.251
1.19
 2      1    2        19.9    22.5    20.3    22.8 -0.401 -0.319   0.0820
0.945
 3      1    3        19.8    21.7    19.9    22.2 -0.121 -0.537  -0.416
1.33
 4      1    4        19.6    21.9    19.9    22.3 -0.284 -0.366  -0.0819
1.06
 5      0.75 1        19.8    22.2    20.0    22.1 -0.244  0.124   0.368
0.775
 6      0.75 2        19.9    22.7    20.3    22.4 -0.401  0.310   0.711
0.611
 7      0.75 3        19.8    22.5    19.9    22.4 -0.121  0.105   0.226
0.855
 8      0.75 4        19.6    22.1    19.9    22.2 -0.284 -0.0585  0.225
0.855
 9      0.5  1        19.8    22.6    20.0    21.7 -0.244  0.847   1.09
0.469
10      0.5  2        19.9    22.9    20.3    22.0 -0.401  0.899   1.30
0.406
# ... with 30 more rows
```

Maximum likelihood estimation on the Cq data taken from samples with known R ratios.

1. Estimation of experimental parameters when the dataset was given in "unpaired" (i.e., long) format

Unpaired Cq dataset with known allele mixing ratios are analyzed with `knownqpcr_unpaired()` function.

```
result <- knownqpcr_unpaired(   Digest=mite.unpaired$Digest,
Gene=mite.unpaired$Gene,
                                trueY=mite.unpaired$FreqTrue,
                                Cq=mite.unpaired$Cq, A=rep(1.0, nrow
(mite.unpaired)),
                                method="BFGS", trace=1, report=10   )
```

```
initial  value 526.625717
iter  10 value 105.043851
iter  20 value 47.820456
iter  30 value -1.189728
final  value -1.601386
converged

Maximum-likelihood estimates with the two-sided 95% CIs
                                          Estimate         2.5%        97.5%
meanDNA (DNA content per unit)        1.255539e-06 7.721917e-07 2.041435e-06
targetScale (rel. target content)     1.170129e+00 1.069274e+00 1.280497e+00
baseChange (after digestion)          2.360702e-01 2.040327e-01 2.731381e-01
SD (Cq measurement error)             2.376223e-01 2.049847e-01 2.754564e-01
zeroAmount (S-target after digestion) 1.564246e-03 1.196945e-03 2.044261e-03
EPCR (PCR multiplification EPCRicacy) 9.712061e-01 9.231223e-01 1.021795e+00
```

2. Estimation of experimental parameters using the "paired" (i.e., wide) format dataset, allowing NAs

Paired Cq dataset with known allele mixing ratios are analyzed with `knownqpcr()` function.

Although the undigested samples were quantified using qPCR only when `FreqTrue == 1`, the "knownqpcr" function (>= v0.3.3) can deal with NA in the Cq data vectors (`housek0`, `target0`, `housek1`, and `target1`). By contrast, no missing values are allowed for `A` (relative DNA contents between samples) and `trueY` (exact allele frequency).

```
result <- knownqpcr(housek0=mite.paired$housek0,
target0=mite.paired$target0,
                    housek1=mite.paired$housek1,
target1=mite.paired$target1,
                    trueY=mite.paired$FreqTrue, A=rep(1.0, nrow
(mite.paired)),
                    method="BFGS", trace=1, report=10)
```

```
initial  value 526.625717
iter  10 value 105.043850
iter  20 value 47.820441
iter  30 value -1.189728
final  value -1.601386
converged

Maximum-likelihood estimates with the two-sided 95% CIs
                                          Estimate         2.5%        97.5%
meanDNA (DNA content per unit)        1.255539e-06 7.721917e-07 2.041435e-06
targetScale (rel. target content)     1.170129e+00 1.069274e+00 1.280497e+00
baseChange (after digestion)          2.360702e-01 2.040327e-01 2.731381e-01
SD (Cq measurement error)             2.376223e-01 2.049847e-01 2.754564e-01
zeroAmount (S-target after digestion) 1.564246e-03 1.196945e-03 2.044261e-03
EPCR (PCR multiplification EPCRicacy) 9.712061e-01 9.231223e-01 1.021795e+00
```

2'. Estimation of experimental parameters when the dataset was given in "paired" format, recycling the Cq values

`mite.paired.recycled` is the paired dataset, where the "undigested" Cq values other than `FreqTrue == 1` were copied from the corresponding replicates of `FreqTrue == 1`. Such data can also be analyzed with `knownqpcr()` function.

```
result <- knownqpcr(housek0=mite.paired.recycled$housek0,
target0=mite.paired.recycled$target0,
                    housek1=mite.paired.recycled$housek1,
target1=mite.paired.recycled$target1,
                    trueY=mite.paired.recycled$FreqTrue, A=rep(1.0, nrow
(mite.paired.recycled)),
                    method="BFGS", trace=1, report=10)
```

```
initial  value 811.268282
iter  10 value 189.797299
iter  20 value -5.694398
iter  30 value -26.773217
final  value -26.780544
converged

Maximum-likelihood estimates with the two-sided 95% CIs
                                        Estimate         2.5%        97.5%
meanDNA (DNA content per unit)        1.301427e-06 9.020759e-07 1.877571e-06
targetScale (rel. target content)     1.184654e+00 1.128954e+00 1.243103e+00
baseChange (after digestion)          2.379246e-01 2.203774e-01 2.568690e-01
SD (Cq measurement error)             2.046781e-01 1.834374e-01 2.283784e-01
zeroAmount (S-target after digestion) 1.583286e-03 1.267953e-03 1.977040e-03
EPCR (PCR multiplification EPCRicacy) 9.670477e-01 9.308465e-01 1.004657e+00
```

Comparison of the estimates: "paired, recycled" versus "paired, allowing NA".

```
meanDNA     1.30e-6     1.26e-6
targetScale 1.18        1.17
baseChange  0.238       0.236
SD          0.205       0.238
zeroAmount  1.58e-3     1.56e-3
EPCR        0.967       0.971
```

You can see both results are almost same, but SD was underestimated when the Cq data are recycled. As the original experimental design is "paired, allowing NA" in Osakabe et al. (2017), the parameter values calculated by

```
knownqpcr(  housek0=mite.paired$housek0, target0=mite.paired$target0,
            housek1=mite.paired$housek1, target1=mite.paired$target1,
            trueY=mite.paired$FreqTrue, A=rep(1.0, nrow(mite.paired))  )
```

are used hereafter. That is,

```
meanDNA     1.26e-6
targetScale 1.17
baseChange  0.236
SD          0.238
zeroAmount  1.56e-3
EPCR        0.971
```

> In the next section, however, `DeltaDeltaCq` and `FreqCalc1971` are calculated on 2' "paird, recycled" dataset because the calculation of each ΔΔCq value needs complete observation of the four condition.

Show the relationship between the allele-mixing ratios and their Cq and ΔΔCq values (Experiment 1 and Figure 3)

First, make the dataset including the calculated values of allele frequency based on `EPCR = 0.971`.

```
mite <- mite.paired.recycled %>%
    dplyr::mutate(FreqCalc1971=1.971^-deldel)
```

Test the heteroskedasticity around ΔΔCq

```
# predictor in the scale of Cq
funcdeldel <- function (p, z=1.56e-03, EPCR=0.971) {
    -log(z*(1-p)+p)/log(1.0+EPCR)
}

# predictor in the scale of raw frequency
funcfreq <- function (p, z=1.56e-03) {
    z*(1-p)+p
}
```

```
# Breusch-Pagan Test (function bptest) to test the heteroskedasticity
library(lmtest)
deldel.predictor <- funcdeldel(mite$FreqTrue, z=1.56e-03, EPCR=0.971)
freq.predictor <- funcfreq(mite$FreqTrue, z=1.56e-03)
deldel.lm <- lm(mite$deldel~deldel.predictor)
freq.lm <- lm(mite$FreqCalc1971~freq.predictor)
summary(deldel.lm)
summary(freq.lm)
lmtest::bptest(deldel.lm)
lmtest::bptest(freq.lm)
```

```
> summary(deldel.lm)

Call:
lm(formula = mite$deldel ~ deldel.predictor)

Residuals:
     Min       1Q   Median       3Q      Max 
-0.60236 -0.19273 -0.00404  0.17686  0.61287 

Coefficients:
                 Estimate Std. Error t value Pr(>|t|)    
(Intercept)      -0.07694    0.07252  -1.061    0.295    
deldel.predictor  1.02518    0.01319  77.703   <2e-16 ***
---
Signif. codes:  0 ‘***’ 0.001 ‘**’ 0.01 ‘*’ 0.05 ‘.’ 0.1 ‘ ’ 1

Residual standard error: 0.28 on 38 degrees of freedom
Multiple R-squared:  0.9937,    Adjusted R-squared:  0.9936 
F-statistic:  6038 on 1 and 38 DF,  p-value: < 2.2e-16
```

```
> summary(freq.lm)

Call:
lm(formula = mite$FreqCalc1971 ~ freq.predictor)

Residuals:
      Min        1Q    Median        3Q       Max 
-0.193350 -0.010329  0.007903  0.009899  0.242789 

Coefficients:
                Estimate Std. Error t value Pr(>|t|)    
(Intercept)    -0.008625   0.013154  -0.656    0.516    
```

```
freq.predictor  1.091599   0.030257  36.078   <2e-16 ***
---
Signif. codes:  0 ‘***’ 0.001 ‘**’ 0.01 ‘*’ 0.05 ‘.’ 0.1 ‘ ’ 1

Residual standard error: 0.06555 on 38 degrees of freedom
Multiple R-squared:  0.9716,    Adjusted R-squared:  0.9709
F-statistic:  1302 on 1 and 38 DF,  p-value: < 2.2e-16
```

```
> lmtest::bptest(deldel.lm)

        studentized Breusch-Pagan test

data:  deldel.lm
BP = 3.1577, df = 1, p-value = 0.07557
```

```
> lmtest::bptest(freq.lm)

        studentized Breusch-Pagan test

data:  freq.lm
BP = 13.978, df = 1, p-value = 0.0001849
```

Code for Figure 3

Error structure around the measured Cq values.

```
deldel.unc <- function (p, scaleDNA=1.26e-06, zeroAmount=1.56e-03,
EPCR=9.71e-01, trans=FALSE) {
    xR <- p*scaleDNA
    xS <- (1-p)*scaleDNA
    deldel <- -log((zeroAmount*xS+xR)/(xS+xR))/log(1+EPCR) # Eq. 8
    if (trans==FALSE) {
        ans <- deldel # As the scale of an expected Cq measure
    } else {
        ans <- (1+EPCR)^-deldel # As the transformation to the allele
frequency
    }
    return(ans)
}
```

```
library(ggplot2)
library(patchwork)
LIM01 <- c(0, 1)
LIMY <- c(-1, 11)

p1 <- ggplot(mite) +
    stat_function(  fun=deldel.unc,
                    args=list(  scaleDNA=1.26e-06, zeroAmount=1.56e-03,
EPCR=9.71e-01,
                                trans=FALSE  ),
                    linetype=1, size=0.3   ) +
    geom_point(aes(y=deldel, x=FreqTrue), shape=1, size=1.0) +
    theme_classic(base_size=10) +
    coord_fixed(ratio=0.1) +
    scale_x_continuous(expand=c(0.02, 0.02), limit=LIM01, breaks=c(0, 0.5,
1)) +
    scale_y_continuous(expand=c(0, 0), limit=LIMY, breaks=seq(0, 10, by=2))
+
    labs( title="A", x="", y=expression(paste(italic(Delta), italic(Delta),
"Cq", sep="")) )

p2 <- ggplot(mite) +
    stat_function(  fun=deldel.unc,
                    args=list(  scaleDNA=1.26e-06, zeroAmount=1.56e-03,
EPCR=9.71e-01,
                                trans=TRUE  ),
                    linetype=1, size=0.3   ) +
    geom_point(aes(y=FreqCalc1971, x=FreqTrue), shape=1, size=1.0) +
    theme_classic(base_size=10) +
    coord_fixed(ratio=1) +
    scale_x_continuous(expand=c(0.02, 0.02), limit=LIM01, breaks=c(0, 0.5,
1)) +
    scale_y_continuous(expand=c(0, 0), limit=c(-0.05, 1.35), breaks=seq(0,
1.3, by=0.2)) +
    labs( title="B",
          x=expression("R allele frequency"),
          y=expression(paste("Frequency calculated from ", italic(Delta),
italic(Delta), "Cq", sep="")) )

p <- p1 / p2
ggsave(file="Figure3.pdf", device=cairo_pdf, plot=p, dpi=300, width=7/2.54,
height=14/2.54)
ggsave(file="Figure3.eps", device=cairo_ps, plot=p, dpi=300, width=7/2.54,
height=14/2.54)
ggsave(file="Figure3.png", type="cairo", plot=p, dpi=300, width=7/2.54,
height=14/2.54)

# save as the enhanced metafile
```

```
library(devEMF)
emf(file="Figure3.emf", width=7/2.54, height=14/2.54, bg="transparent",
pointsize=10, family="Arial", coordDPI=300)
p
dev.off()
```

Part II freqpcr: function of simultaneous parameter estimation

First, make a dummy dataset for demonstration.

```
Dataset for freqpcr() is always prepared in the "paired" format. Missing Cq
values are allowed, and no effect on the estimation result. The size vector
of the bulk sample, N, must not contain missing values.
```

```
library(freqpcr); packageVersion("freqpcr");
P <- 0.15
K <- 4
ntrap <- 4 # ntrap must be >= 1
npertrap <- 8 # npertrap must be >= 1
scaleDNA <- (1/K)*1e-06
targetScale <- 1.2
baseChange <- 0.2
EPCR <- 0.97
zeroAmount <- 1.6e-03
sdMeasure <- 0.2

# Make a dummy dataset (haploid)
dat.DNA <- make_dummy(  rand.seed=71, P, K, ntrap, npertrap, scaleDNA,
                        targetScale, baseChange, EPCR, zeroAmount,
sdMeasure, diploid=FALSE  )
dat.DNA
(dat.gamma <- data.frame(   N=dat.DNA@N, housek0=dat.DNA@housek0,
target0=dat.DNA@target0,
                            housek1=dat.DNA@housek1,
target1=dat.DNA@target1,
                            RFreqMeasure=dat.DNA@RFreqMeasure,
ObsP=dat.DNA@ObsP   ))

# Make a dummy dataset (diploid)
```

```
dat2.DNA <- make_dummy( rand.seed=71, P, K, ntrap, npertrap, scaleDNA,
                        targetScale, baseChange, EPCR, zeroAmount,
sdMeasure, diploid=TRUE )
dat2.DNA
(dat2.gamma <- data.frame(  N=dat2.DNA@N, housek0=dat2.DNA@housek0,
target0=dat2.DNA@target0,
                            housek1=dat2.DNA@housek1,
target1=dat2.DNA@target1,
                            RFreqMeasure=dat2.DNA@RFreqMeasure,
ObsP=dat2.DNA@ObsP  ))
```

Dummy Cq data object class (haploid)

```
> dat.DNA
An object of class "CqList"
Slot "N":
[1] 8 8 8 8

Slot "m":
     [,1] [,2] [,3] [,4]
[1,]    1    1    1    0
[2,]    7    7    7    8

Slot "xR":
[1] 6.654827e-07 1.540772e-06 7.998461e-07 0.000000e+00

Slot "xS":
[1] 5.541084e-06 7.909241e-06 8.052976e-06 1.004673e-05

Slot "housek0":
[1] 17.57120 16.90864 17.14117 16.88547

Slot "target0":
[1] 17.65665 16.84725 16.58306 16.90106

Slot "housek1":
[1] 19.90382 19.08542 19.59406 19.42896

Slot "target1":
[1] 23.13257 21.59854 22.97203 28.34278

Slot "DCW":
[1]  0.08544953 -0.06139074 -0.55811003  0.01559466

Slot "DCD":
```

```
[1] 3.228745 2.513126 3.377968 8.913814

Slot "deldel":
[1] 3.143296 2.574517 3.936078 8.898219

Slot "RFreqMeasure":
[1] 0.11868765 0.17453873 0.06933585 0.00239759

Slot "ObsP":
[1] 0.11868765 0.17453873 0.06933585 0.00239759

Slot "rand.seed":
[1] 71
```

Dummy Cq data (haploid): qPCR observation data are usually prepared in this format.

```
> (dat.gamma <- data.frame(   N=dat.DNA@N, housek0=dat.DNA@housek0,
target0=dat.DNA@target0,
+                             housek1=dat.DNA@housek1,
target1=dat.DNA@target1,
+                             RFreqMeasure=dat.DNA@RFreqMeasure,
ObsP=dat.DNA@ObsP   ))
  N  housek0  target0  housek1  target1 RFreqMeasure       ObsP
1 8 17.57120 17.65665 19.90382 23.13257   0.11868765 0.11868765
2 8 16.90864 16.84725 19.08542 21.59854   0.17453873 0.17453873
3 8 17.14117 16.58306 19.59406 22.97203   0.06933585 0.06933585
4 8 16.88547 16.90106 19.42896 28.34278   0.00239759 0.00239759
```

Dummy Cq data object class (diploid)

```
> dat2.DNA
An object of class "CqList"
Slot "N":
[1] 8 8 8 8

Slot "m":
     [,1] [,2] [,3] [,4]
[1,]    0    0    0    0
[2,]    2    1    4    4
[3,]    6    7    4    4

Slot "xR":
[1] 1.599798e-06 1.249008e-06 4.770976e-06 3.342741e-06
```

```
Slot "xS":
[1] 1.513851e-05 1.706749e-05 1.564665e-05 9.480615e-06

Slot "housek0":
[1] 16.13263 16.32947 15.97995 16.30548

Slot "target0":
[1] 16.14822 15.66516 15.30663 16.40500

Slot "housek1":
[1] 18.67612 18.53178 18.06831 19.17237

Slot "target1":
[1] 21.53573 22.12281 20.64181 20.75895

Slot "DCW":
[1]  0.01559466 -0.66430459 -0.67331097  0.09952126

Slot "DCD":
[1] 2.859606 3.591031 2.573500 1.586582

Slot "deldel":
[1] 2.844011 4.255336 3.246811 1.487061

Slot "RFreqMeasure":
[1] 0.14539009 0.05584025 0.11064299 0.36484699

Slot "ObsP":
[1] 0.14539009 0.05584025 0.11064299 0.36484699

Slot "rand.seed":
[1] 71
```

Dummy Cq data (diploid): qPCR observation data are usually prepared in this format.

```
> (dat2.gamma <- data.frame(  N=dat2.DNA@N, housek0=dat2.DNA@housek0,
target0=dat2.DNA@target0,
+                             housek1=dat2.DNA@housek1,
target1=dat2.DNA@target1,
+                             RFreqMeasure=dat2.DNA@RFreqMeasure,
ObsP=dat2.DNA@ObsP  ))
  N  housek0  target0  housek1  target1 RFreqMeasure       ObsP
1 8 16.13263 16.14822 18.67612 21.53573   0.14539009 0.14539009
2 8 16.32947 15.66516 18.53178 22.12281   0.05584025 0.05584025
```

```
3 8 15.97995 15.30663 18.06831 20.64181   0.11064299 0.11064299
4 8 16.30548 16.40500 19.17237 20.75895   0.36484699 0.36484699
```

Log likelihood

Log likelihood defined on the gamma distribution model

```
system.time(
hoge <- freqpcr:::.freqpcr_loglike( X=c(P=-2, K=1, targetScale=0,
sdMeasure=log(0.24), EPCR=0),
                                    N=dat.gamma$N, DCW=dat.gamma$target0-
dat.gamma$housek0,
                                    DCD=dat.gamma$target1-dat.gamma$housek1,
                                    zeroAmount=0.0016, para.fixed=NULL,
beta=FALSE, diploid=FALSE )
)
hoge
```

```
> hoge
[1] 7.049412
```

Log likelihood defined on the beta distribution model

```
system.time(
hoge <- freqpcr:::.freqpcr_loglike( X=c(P=-2, K=1, targetScale=0,
sdMeasure=log(0.24), EPCR=0),
                                    N=dat.gamma$N, DCW=dat.gamma$target0-
dat.gamma$housek0,
                                    DCD=dat.gamma$target1-dat.gamma$housek1,
                                    zeroAmount=0.0016, para.fixed=NULL,
beta=TRUE, diploid=FALSE )
)
hoge
```

```
> hoge
[1] 7.049006
```

Simultaneous parameter estimation with `freqpcr()` function

1. Assmmsing the gamma distributions and all parameters other than EPCR are unknown

If you feed some value for each of `P`, `K`, `targetScale`, `sdMeasure`, it is then treated as a fixed parameter value and not estimated via `freqpcr()` function.

If a parameter is unknown, put `NULL` or simply ignore them. However, if you want to treat `EPCR` as unknown variable, you must explicitly write as `EPCR=NULL`, or it is treated as a fixed parameter with default (= 0.99).

```
EPCR <- 0.97
result1 <- freqpcr( N=dat.gamma$N,
                    housek0=dat.gamma$housek0, target0=dat.gamma$target0,
                    housek1=dat.gamma$housek1, target1=dat.gamma$target1,
                    P=NULL, K=NULL, targetScale=NULL, sdMeasure=NULL,
EPCR=EPCR,
                    zeroAmount=1.6e-03, beta=FALSE, diploid=FALSE,
                    pvalue=0.05, gradtol=1e-4, steptol=1e-9, iterlim=100,
maxtime=600, print.level=1)
result1
```

```
print.level
0: for no output during calculation
1: for first/last iterations
2: for all iterations
```

```
> result1
An object of class "CqFreq"
Slot "report":
                                               Estimate Fixed   (scaled)
(scaled.SE)       2.5%     97.5%
P (R-allele frequency)                        0.1141011     0 -2.0495184
0.58284394 0.03947276 0.2875803
K (gamma shape parameter)                     3.5678195     0
1.2719546         NaN        NaN        NaN
targetScale (relative amount of target locus) 1.1481927     0
0.1381891         NaN        NaN        NaN
```

```
Cq measurement error (SD)                     0.2187155     0 -1.5199833
0.06417172 0.19286641 0.2480291
EPCR (Duplication efficiency of PCR)          0.9700000     1
NA           NA          NA         NA

Slot "obj":
$minimum
[1] 6.808096

$estimate
[1] -2.0495184  1.2719546  0.1381891 -1.5199833

$gradient
[1]  2.704796e-01  7.025499e+04  8.934158e+04 -5.877667e+04

$hessian
              [,1]          [,2]          [,3]          [,4]
[1,]     2.9197547 -1.847117e+03 -2.345098e+03 -1.581763e-01
[2,] -1847.1167828 -1.104395e+09 -1.404740e+09 -2.500117e+04
[3,] -2345.0977397 -1.404740e+09 -1.786766e+09 -3.168260e+04
[4,]    -0.1581763 -2.500117e+04 -3.168260e+04  9.021054e+00

$code
[1] 3

$iterations
[1] 29


Slot "cal.time":
   user  system elapsed
  11.44    0.65   12.14
```

2. Simultaneous parameter estimation with beta distribution

To use the Beta model setting, run `freqpcr()` with `beta=TRUE`. This is the default of `freqpcr()` function.

First, all parameters other than EPCR are set unknown.

```
EPCR <- 0.97
result1 <- freqpcr( N=dat.gamma$N,
                    housek0=dat.gamma$housek0, target0=dat.gamma$target0,
                    housek1=dat.gamma$housek1, target1=dat.gamma$target1,
                    P=NULL, K=NULL, targetScale=NULL, sdMeasure=NULL,
```

```
EPCR=EPCR,
                    zeroAmount=1.6e-03, beta=TRUE, diploid=FALSE,
                    pvalue=0.05, gradtol=1e-4, steptol=1e-9, iterlim=100,
maxtime=600, print.level=2)
result1
```

```
> result1
An object of class "CqFreq"
Slot "report":
                                                  Estimate Fixed  (scaled)
(scaled.SE)       2.5%       97.5%
P (R-allele frequency)                          0.09773189     0 -2.222684
0.60914921 0.03178086   0.2633220
K (gamma shape parameter)                      20.92728468     0  3.041054
1.83522498 0.57354375 763.5882087
targetScale (relative amount of target locus)  1.11922896     0  0.112640
0.08911953 0.93985371   1.3328388
Cq measurement error (SD)                       0.20973065     0 -1.561931
0.32845068 0.11017528   0.3992451
EPCR (Duplication efficiency of PCR)            0.97000000     1
NA          NA          NA          NA

Slot "obj":
$minimum
[1] 6.094915

$estimate
[1] -2.222684  3.041054  0.112640 -1.561931

$gradient
[1] -3.400973e-05 -8.275977e-05 -5.170087e-05  8.878366e-05

$hessian
            [,1]        [,2]       [,3]       [,4]
[1,]  2.71023737  0.05094365   1.168535 -0.1766755
[2,]  0.05094365  0.37630061   2.045198 -0.6539722
[3,]  1.16853469  2.04519835 147.389559  6.4578191
[4,] -0.17667547 -0.65397222   6.457819 11.1504638

$code
[1] 1

$iterations
[1] 12
```

```
Slot "cal.time":
   user  system elapsed 
   0.73    0.20    0.94 
```

Faster, but `K` was overestimated.

2. `freqpcr()` assuming `K=2` (true gamma shape is known)

```
EPCR <- 0.97
result1 <- freqpcr( N=dat.gamma$N,
                    housek0=dat.gamma$housek0, target0=dat.gamma$target0,
                    housek1=dat.gamma$housek1, target1=dat.gamma$target1,
                    P=NULL, K=4, targetScale=NULL, sdMeasure=NULL, 
EPCR=EPCR,
                    zeroAmount=1.6e-03, beta=TRUE, diploid=FALSE,
                    pvalue=0.05, gradtol=1e-4, steptol=1e-9, iterlim=100, 
maxtime=600, print.level=1)
result1
```

```
> result1
An object of class "CqFreq"
Slot "report":
                                           Estimate Fixed   (scaled) 
(scaled.SE)       2.5%     97.5%
P (R-allele frequency)                    0.1035739     0 -2.1581308 
0.60872713 0.03385543 0.2758687
K (gamma shape parameter)                 4.0000000     1         
NA          NA         NA         NA
targetScale (relative amount of target locus) 1.1400313     0  0.1310557 
0.08538882 0.96434774 1.3477206
Cq measurement error (SD)                 0.2025401     0 -1.5968176 
0.30962382 0.11039736 0.3715893
EPCR (Duplication efficiency of PCR)      0.9700000     1         
NA          NA         NA         NA

Slot "obj":
$minimum
[1] 6.678446

$estimate
[1] -2.1581308  0.1310557 -1.5968176
```

```
$gradient
[1] -7.381558e-06 -5.636824e-05  1.196814e-05

$hessian
           [,1]       [,2]       [,3]
[1,]  2.7089129   1.038771 -0.1281927
[2,]  1.0387709 139.379620  4.3497087
[3,] -0.1281927   4.349709 10.5764221

$code
[1] 1

$iterations
[1] 9


Slot "cal.time":
   user  system elapsed
   0.44    0.13    0.56
```

Pretty fast and accurate. If the size of the shape parameter `K` is known, this option is the best.

Tweaks

Since `freqpcr v0.3.2`, you can input Cq data with missing values (results are not shown)

```
#dat.gamma[1, "target1"] <- NA
#dat.gamma[1, "housek0"] <- NA
```

Since freqpcr v0.3.2, you can use a "continuous" sample allele-ratio distribution. This extended model is under development and not used in the paper.

In the continuous model, the environmental allele ratio (p) follows directly a beta/gamma distribution, skipping the binomial sampling process.

These direct DNA sampling may be consistent with environmental DNA though it has not been verified in field.

```
# Log likelihood by the gamma distribution model, ignoring binomial sampling
(continuous gamma model)
system.time(
hoge <- freqpcr:::.freqpcr_loglike_cont(X=c(P=-2, K=1, targetScale=0,
sdMeasure=log(0.24), EPCR=0),
                                        A=dat.gamma$N,
                                        DCW=dat.gamma$target0-
dat.gamma$housek0,
                                        DCD=dat.gamma$target1-
dat.gamma$housek1,
                                        zeroAmount=0.0016, para.fixed=NULL,
beta=FALSE)
)
hoge
```

```
> hoge
[1] 86.18858
```

```
# Log likelihood by the beta distribution model, ignoring binomial sampling
(continuous beta model)
system.time(
hoge <- freqpcr:::.freqpcr_loglike_cont(X=c(P=-2, K=1, targetScale=0,
sdMeasure=log(0.24), EPCR=0),
                                        A=dat.gamma$N,
                                        DCW=dat.gamma$target0-
dat.gamma$housek0,
                                        DCD=dat.gamma$target1-
dat.gamma$housek1,
                                        zeroAmount=0.0016, para.fixed=NULL,
beta=TRUE)
)
hoge
```

```
> hoge
[1] 14.08126
```

Function of parameter estimation from seed

`sim_dummy()`, a wrapper of `freqpcr()`, adopts the result of `make_dummy()` and returns the estimates of p and other parameters.

> Note that a `CqList` class object, the output of `make_dummy()`, in no way contains the value of the original parameters like p. The function is thus used for the evaluation of `freqpcr()` (Experiment 2 appearing in ESM3).

```
dat.DNA
EPCR <- 0.97
zeroAmount <- 0.0016
result <- sim_dummy(CqList=dat.DNA, EPCR=EPCR, zeroAmount=zeroAmount,
                    K=K, # only if some of the parameters e.g. K is given a
priori
                    beta=TRUE, diploid=FALSE, maxtime=60, print.level=1,
aux="Beta TRUE")
result
```

```
> result
An object of class "CqFreq"
Slot "report":
                                               Estimate Fixed   (scaled)
(scaled.SE)       2.5%     97.5%
P (R-allele frequency)                        0.1035739     0 -2.1581308
0.60872713 0.03385543 0.2758687
K (gamma shape parameter)                     4.0000000     1
NA          NA          NA         NA
targetScale (relative amount of target locus) 1.1400313     0  0.1310557
0.08538882 0.96434774 1.3477206
Cq measurement error (SD)                     0.2025401     0 -1.5968176
0.30962382 0.11039736 0.3715893
EPCR (Duplication efficiency of PCR)          0.9700000     1
NA          NA          NA         NA

Slot "obj":
$minimum
[1] 6.678446

$estimate
[1] -2.1581308  0.1310557 -1.5968176

$gradient
[1] -7.381558e-06 -5.636824e-05  1.196814e-05

$hessian
```

```
              [,1]       [,2]       [,3]
[1,]  2.7089129   1.038771 -0.1281927
[2,]  1.0387709 139.379620  4.3497087
[3,] -0.1281927   4.349709 10.5764221

$code
[1] 1

$iterations
[1] 9


Slot "cal.time":
   user  system elapsed
   0.40    0.07    0.49
```

Case of diploidy

`freqpcr()` also deals with diploidy (`diploid=TRUE`). This is an experimental implementation. We assume i.i.d. on the DNA quantities of S and R owned by heterozygotes. Both `beta=TRUE/FALSE` can be used.

```
system.time(
hoge <- freqpcr:::.freqpcr_loglike( X=c(P=-2, K=1, targetScale=0,
sdMeasure=log(0.24), EPCR=0),
                                    N=dat2.gamma$N,
                                    DCW=dat2.gamma$target0-
dat2.gamma$housek0,
                                    DCD=dat2.gamma$target1-
dat2.gamma$housek1,
                                    zeroAmount=0.0016, para.fixed=NULL,
beta=FALSE, diploid=TRUE )
)
hoge
```

```
> hoge
[1] 9.009644
```

Simultaneous parameter estimation for diploids are as follows.

Diploid, assuming i.i.d, use beta distribution, exact size of K is known

```
EPCR <- 0.97
result1 <- freqpcr( N=dat2.gamma$N,
                    housek0=dat2.gamma$housek0, target0=dat2.gamma$target0,
                    housek1=dat2.gamma$housek1, target1=dat2.gamma$target1,
                    P=NULL, K=4, targetScale=NULL, sdMeasure=NULL,
EPCR=EPCR,
                    zeroAmount=1.6e-03, beta=TRUE, diploid=TRUE,
                    pvalue=0.05, gradtol=1e-4, steptol=1e-9, iterlim=100,
maxtime=600, print.level=1)
result1
```

```
> result1
An object of class "CqFreq"
Slot "report":
                                               Estimate Fixed   (scaled)
(scaled.SE)       2.5%     97.5%
P (R-allele frequency)                        0.1581764     0 -1.6718598
0.4275540 0.07517052 0.3028276
K (gamma shape parameter)                     4.0000000     1
NA          NA          NA         NA
targetScale (relative amount of target locus) 1.2088993     0  0.1897103
0.1209760 0.95370738 1.5323752
Cq measurement error (SD)                     0.2526398     0 -1.3757906
0.3402709 0.12967689 0.4921992
EPCR (Duplication efficiency of PCR)          0.9700000     1
NA          NA          NA         NA

Slot "obj":
$minimum
[1] 6.791649

$estimate
[1] -1.6718598  0.1897103 -1.3757906

$gradient
[1] 0.0001637775 0.0006179155 0.0001780152

$hessian
           [,1]      [,2]       [,3]
[1,]  6.0753247  6.669574 -0.2452993
[2,]  6.6695739 76.380224  2.2547991
[3,] -0.2452993  2.254799  8.7389088
```

```
$code
[1] 1

$iterations
[1] 8


Slot "cal.time":
   user  system elapsed
   0.81    0.13    0.94
```

Diploid, use gamma distribution and K is known.

> Caution: the combination of beta=FALSE (the gamma model) and diploid=TRUE is very slow!

```
result1 <- freqpcr( N=dat2.gamma$N,
                    housek0=dat2.gamma$housek0, target0=dat2.gamma$target0,
                    housek1=dat2.gamma$housek1, target1=dat2.gamma$target1,
                    P=NULL, K=4, targetScale=NULL, sdMeasure=NULL,
EPCR=0.97,
                    zeroAmount=1.6e-03, beta=FALSE, diploid=TRUE,
                    pvalue=0.05, gradtol=1e-4, steptol=1e-9, iterlim=100,
maxtime=600, print.level=2)
result1
```

```
> result1
An object of class "CqFreq"
Slot "report":
                                              Estimate Fixed   (scaled)
(scaled.SE)       2.5%     97.5%
P (R-allele frequency)                       0.1499250     0 -1.7351892
0.4224756 0.07154295 0.2875829
K (gamma shape parameter)                    4.0000000     1
NA          NA         NA         NA
targetScale (relative amount of target locus) 1.2011939    0
0.1833159        NaN        NaN        NaN
Cq measurement error (SD)                    0.2940397     0 -1.2240404
0.2615032 0.17612220 0.4909055
EPCR (Duplication efficiency of PCR)         0.9700000     1
NA          NA         NA         NA
```

```
Slot "obj":
$minimum
[1] 6.899955

$estimate
[1] -1.7351892  0.1833159 -1.2240404

$gradient
[1] -4.424957e-01  8.848356e+04 -7.228854e+04

$hessian
              [,1]          [,2]          [,3]
[1,]  5.516096e+00 -1.536161e+04 -2.011408e-01
[2,] -1.536161e+04 -1.769565e+09 -1.188202e+05
[3,] -2.011408e-01 -1.188202e+05  6.766996e+00

$code
[1] 3

$iterations
[1] 36


Slot "cal.time":
   user  system elapsed
 162.90    1.19  164.25
```

sim_dummy() in case of diploidy

```
result <- sim_dummy(CqList=dat2.DNA, EPCR=EPCR, zeroAmount=zeroAmount,
                    K=K, # only if some of the parameters e.g. K is given a
priori
                    beta=TRUE, diploid=TRUE, maxtime=60, print.level=1,
aux="just a test")
result
```

If you specified `diploid=TRUE` for the dataset generated as haploids...

```
result <- sim_dummy(CqList=dat.DNA, EPCR=EPCR, zeroAmount=zeroAmount,
                    K=K, # only if some of the parameters e.g. K is given a
```

```
priori
                    beta=TRUE, diploid=TRUE, maxtime=60, print.level=1,
aux="just a test")
result
```

Actually, not bad.

If you specified `diploid=FALSE` for the dataset generated as diploids...

```
result <- sim_dummy(CqList=dat2.DNA, EPCR=EPCR, zeroAmount=zeroAmount,
                    K=K, # only if some of the parameters e.g. K is given a
priori
                    beta=TRUE, diploid=FALSE, maxtime=60, print.level=1,
aux="just a test")
result
```

`p` is strongly overestimated in this case.
