# Supplementary material for: freqpcr: Estimation of population allele frequency using qPCR ΔΔCq measures from bulk samples
Source: Mol Ecol Resour. 2021 Dec 9;22(4):1380–93. doi: 10.1111/1755-0998.13554 (PMC9300209; doi:10.1111/1755-0998.13554)
Supplement: Supplementary file 4 — Appendix S4 [file MEN-22-1380-s004.pdf]

freqpcr: estimation of population allele frequency using qPCR ΔΔCq measures from bulk samples

Masaaki Sudo, Masahiro Osakabe

ORCID ID:

- 0000-0001-9834-9857 (Masaaki Sudo)
- 0000-0002-2246-3431 (Masahiro Osakabe)

```
R package name: freqpcr
Short description: interval estimation of population allele frequency based
on ΔΔCq measures of quantitative PCR over bulk samples
Package author & maintainer: Masaaki Sudo (NARO, JAPAN)
https://github.com/sudoms/freqpcr
```

ESM 3

R source code for the numerical simulation (Experiment 2) and the codes for Figures 4 and after.

Part III Numerical experiment to evaluate the functions for simultaneous parameter estimation

For each of the 624 parameter regions, the estimation on a randomly-generated dataset was replicated 1000 times. The dummy datasets for the n-th replicate were generated using `freqpcr::make_dummy()` with the RNG seed == n.

`(624 regions) * (5 replicates)` were simulated in a batch. This is what called `iter` hereafter.

Therefore, the simulation comprised 200 batches (iterations). Those batches can be run on separate machines.

Furthermore, the procedure was evaluated in four different configurations.

1. Assuming Beta distribution, fixing K = 1 (and P, targetScale, and sdMeasure are unknown).
2. Assuming Beta distribution, treating K (and all other parameters) as unknown.
3. Assuming Gamma distribution, fixing K = 1
4. Assuming Gamma distribution, treating K as unknown.

As the configurations 3 and 4 require more time, they were only replicated 250 times (first 50 batches).

> Note: those simulations were conducted on haploidy. The function also has a limited ability to deal with diploids, but it has not been evaluated in the paper because it requires considerable machine resource.

```
library(tidyverse)
library(parallel)
library(future)
library(furrr) # if not installed, install.packages("furrr")
library(installr) # if not installed, install.packages("installr")
sessionInfo()
library(freqpcr); packageVersion("freqpcr");
options(future.makeNodePSOCK.connectTimeout=30*24*60*60); getOption
("future.makeNodePSOCK.connectTimeout");
options(future.makeNodePSOCK.timeout=24*60*60); getOption
("future.makeNodePSOCK.timeout");

# Set information for the session.
set.seed(NULL)
trialid <- 172308L # rename it as you like
analysis_name <- paste("200824", trialid, sep=".")
#repl.total <- 2; repl.each <- 1; # for small demo
repl.total <- 1000; repl.each <- 5; # real code: number of replicates run in
a single iteration
```

The following code is configured for the first 50 iterations (50 iterations * 5 `repl.each` = 250 replicates).

- For the first time, set "iter.skip <- 0" and "iter.to <- 50" to execute the first 50 iterations.
- For the second time, set "iter.skip <- 50" and "iter.to <- 100" to execute the next 50 iterations.

```
iter.skip <- 0 # skip first n iterations (default 0 = no skip)
#iter.to <- 50 # the last iteration actually calculated
iter.to <- 2 # for small demos; calculate only the first two iterations (624
regions * 10 replicates)
iter <- (iter.skip+1):iter.to # the iterator values which are actually used
in the session.

P <- c(0.01, 0.05, 0.1, 0.25, 0.5, 0.75)
K <- c(1, 3, 9, 27)
ntrap <- 2^seq(0, 6, by=1) # N: we do not consider ntrap=1 (DNA from all
individuals are extracted together)
npertrap <- 2^seq(0, 6, by=1) # n_h: npertrap must be >= 1
diploid <- c(FALSE)

# om: the tibble that stores the parameter combination (and the results).
# regardless of "iter.skip", all rows are generated.
om <- tidyr::expand_grid(P, K, ntrap, npertrap, replicate=c(1:repl.total),
diploid) %>%
    dplyr::mutate(  scaleDNA=(1/K)*1e-06, targetScale=1.2, baseChange=0.24,
                    EPCR=0.97, zeroAmount=1.6e-03, sdMeasure=0.2  ) %>%
    dplyr::mutate(ntotal=ntrap*npertrap) %>%
    dplyr::filter(ntotal<=128, ntotal>=4, ntrap>=2)
om$LID <- c(1:nrow(om))
set.seed(1729)
om$rseed <- sample(nrow(om), replace=FALSE) # rseed is affected by the size
of "repl.total"
set.seed(NULL)
om <- om %>%
    dplyr::filter(replicate > floor(iter.skip*repl.each), replicate <=
ceiling(iter.to*repl.each)) %>%
    dplyr::select(LID, everything())
print(om)
print(nrow(om))
print(xtabs( ~ ntotal + ntrap, data=om))
```

Generate the dummy data of DNA yield for each bulk sample. This operation returns lists in the tibble.

> This process should not be parallelized due to the consistency of RNG seeds. Calculation time is not proportional: 18000 regions -> 17 sec, 78,000 -> 271 sec, 156,000 -> 1668 sec

```
ptime0 <- proc.time()
om <- om %>%
    dplyr::group_by(LID) %>%
```

```
    tidyr::nest() %>%
    dplyr::mutate(  DNA=purrr::map( .x=data,
                                    .f=~make_dummy( rand.seed=.$rseed,
P=.$P, K=.$K,
                                                    ntrap=.$ntrap,
npertrap=.$npertrap, scaleDNA=.$scaleDNA,

targetScale=.$targetScale, baseChange=.$baseChange,
                                                    EPCR=.$EPCR,
zeroAmount=.$zeroAmount, sdMeasure=.$sdMeasure,
                                                    diploid=.$diploid ) )  )
%>%
    dplyr::mutate(  ave_P_mean=purrr::map_dbl(.x=DNA, .f=~mean(pluck(.,
"deldel"))),
                    ave_P_sd=purrr::map_dbl(.x=DNA, .f=~sd(pluck(.,
"deldel")))  ) %>%
    tidyr::unnest(cols=data) %>%
    dplyr::ungroup() %>%
    dplyr::arrange(rseed) %>% # arrange() should comes after mutate(), or
calculation time explodes.
    dplyr::mutate(ave_P_est=(1.0+EPCR)^(-ave_P_mean)) # estimation by simple
averaging of Cq
cal.time <- proc.time()-ptime0; print(cal.time);
invisible(gc()); invisible(gc());
print(om)
```

The resultant data looks like:

```
om[1, "DNA"][[1]][[1]]
```

---

simulation 1. Beta distribution, K = 1

```
session <- "Beta_K1" # variable
param.beta <- TRUE # variable
param.K <- 1 # variable

# the following simulation code will be recycled.
for (i in iter) {
    iter.num <- formatC( as.integer(i), width=4, flag="0" )
    cat(paste( "\nStarting iteration No. ", i, " (min:", iter.skip+1, ",
max: ", iter.to, ")", " / ",
```

```
                ceiling(repl.total/repl.each), "\n\n", sep="" ))
    om.each <- om %>%
        dplyr::filter(replicate > (i-1)*repl.each, replicate <= i*repl.each)
%>%
#        dplyr::filter(npertrap==8) %>% # for small demos
#        dplyr::filter(ntrap==2) %>%
        dplyr::mutate(beta=param.beta, Session=session) # variable
    print(om.each); cat("\n"); print(xtabs( ~ ntrap + npertrap,
data=om.each)); cat("\n");
    flush.console()

    # Initialize log output
    fout <- file(paste("o", analysis_name, session, iter.num, "txt",
sep="."), "w")

    cl <- future::makeClusterPSOCK(availableCores()-1,
connectTimeout=24*60*60, timeout=24*60*60)
    cl.pid <- purrr::map_int(cl, ~pluck(., "session_info", "process",
"pid"))
    cat("Cluster cl was initialized with the following PID:\n"); print
(cl.pid);
    invisible(clusterEvalQ(cl, library(freqpcr))) # load the package on
cluster
    future::plan(cluster, workers=cl);
    cat(paste("furrr: plan cluster, worker = cl, number of workers = ",
nbrOfWorkers(), "\n", sep=""));

    # Estimation with freqpcr()
    ptime0 <- proc.time()
    e1 <- try( {
        cat(paste("Calculation started in ", Sys.time(), "\n", sep=""));
flush.console();
        capture.output( temp <- om.each %>%
            dplyr::group_by(LID) %>%
            tidyr::nest() %>%
            furrr::future_map(  .x=.$data,
                                .f=~sim_dummy(  CqList=pluck(.$DNA, 1),
EPCR=.$EPCR, zeroAmount=.$zeroAmount,
                                                K=param.K, # variable
                                                beta=.$beta,
diploid=.$diploid,
                                                maxtime=120, print.level=1,
# 120
                                                aux=c
(  replicate=.$replicate, rseed=.$rseed,
                                                        P=.$P, K=.$K,
                                                        ntrap=.$ntrap,
npertrap=.$npertrap, beta=.$beta  )  )  ),
```

```
            file=fout, append=FALSE, type=c("output") )
            # file = NULL or /dev/null (unix) to supress the output
        cat(paste("Calculation ended in   ", Sys.time(), "\n", sep=""));
    }, silent=FALSE )
    print(proc.time()-ptime0); flush.console();

    if (class(e1)=="try-error") {
        while (class(e1)=="try-error") {
            cat("All 'Rscript.exe' actually exist on your machine:\n");
            print(installr::get_Rscript_PID());
            cat("Recognized by R host:\n");
            print(purrr::map_int(cl, ~pluck(., "session_info", "process",
"pid")));
            cat("freqpcr() failed in some parameter regions. Retry.\n")
            result <- om.each

            close(fout)
            future::plan(sequential)
            try({parallel::stopCluster(cl)})
            installr::kill_pid(cl.pid)
            closeAllConnections()
            gc(); gc();

            # Re-initialize log output
            fout <- file(paste("o", analysis_name, session, iter.num, "txt",
sep="."), "w")
            # use fewer cluster cores if connection errors occurred.
            cl <- future::makeClusterPSOCK(min(8, floor(availableCores
()/2)), connectTimeout=24*60*60, timeout=24*60*60)
            cl.pid <- purrr::map_int(cl, ~pluck(., "session_info",
"process", "pid"))
            cat("\nCluster cl was re-initialized with the following
PID:\n"); print(cl.pid);
            invisible(clusterEvalQ(cl, library(freqpcr))) # load the package
on cluster
            future::plan(cluster, workers=cl)

            # Recalculation with freqpcr()
            ptime0 <- proc.time()
            e1 <- try( {
                cat(paste("Re-calculation started in ", Sys.time(), "\n",
sep="")); flush.console();
                capture.output( temp <- om.each %>%
                    dplyr::group_by(LID) %>%
                    tidyr::nest() %>%
                    furrr::future_map(  .x=.$data,
                                        .f=~sim_dummy(  CqList=pluck(.$DNA,
1), EPCR=.$EPCR, zeroAmount=.$zeroAmount,
```

```
                                                        K=param.K, #
variable
                                                        beta=.$beta,
diploid=.$diploid,
                                                        maxtime=120,
print.level=1, # 120
                                                        aux=c
(  replicate=.$replicate, rseed=.$rseed,
                                                                P=.$P,
K=.$K,

ntrap=.$ntrap, npertrap=.$npertrap, beta=.$beta  )  )  ),
                    file=fout, append=FALSE, type=c("output") ) # "NUL"
or /dev/null (unix) to supress the output
                cat(paste("Re-calculation ended in   ", Sys.time(), "\n",
sep=""));
            }, silent=FALSE )
            print(proc.time()-ptime0); flush.console();
        }
    }

    # First, close the log file. If fout has not been initialized, this
operation returns error.
    try( {
        close(fout); cat("The log object 'fout' was closed successfully. ");
    } )
    future::plan(sequential);
    cat(paste("Switched to plan sequential, number of workers = ",
nbrOfWorkers(), "\n", sep=""));
    stopcl <- try( {
        parallel::stopCluster(cl); cat("Cluster cl stopped
successfully.\n");
    } )
    # If even a part of cluster nodes has connection failure (when "freqpcr
failed."),
    # stopCluster(cl) returns error such as "Error in serialize(data,
node$con)".
    # Then you can still see the rest of the non-removable nodes with
showConnection().
    # They must be closed.
    if (class(stopcl)=="try-error") {
        cat("'stopCluster(cl)' failed; force 'kill_pid' and
'closeAllConnections()';\n")
        cat("Available connections on R host:\n"); print(showConnections
(all=TRUE));
        installr::kill_pid(cl.pid) # Kill Rscript.exe by PID.
        # You can also kill all "Rscript.exe" but this goes wrong when you
are running multiple R.
```

```
#        installr::kill_process("Rscript")
        cat("Cluster cl was forced to close.\n")
    }
    closeAllConnections() # close connections except for stdin, stdout,
stderr
    rm(stopcl); rm(cl.pid); rm(cl); gc(); gc();

    # recall the rows of "time-over"
    # To reduce idle nodes, "maxtime" set above (120 s) was smaller than the
default (600 s).
    temp.iter <- unlist(purrr::map(.x=temp, .f=~pluck(., "obj",
"iterations", 1)))
    over.LID <- is.na(temp.iter)
    if (sum(over.LID)>0) {
        cat(paste("\n", sum(over.LID), " rows ended in timeover.\n",
sep=""))
        # Reopen log output as append mode.
        fout <- file(paste("o", analysis_name, session, iter.num, "txt",
sep="."), "a")
        ptime0 <- proc.time()
        e1 <- try( {
            cat(paste("Re-calculation for timeover started in ", Sys.time
(), "\n", sep=""));
            flush.console();
            capture.output( temp.over <- om.each %>%
                dplyr::filter(over.LID) %>%
                dplyr::group_by(LID) %>%
                tidyr::nest() %>%
                purrr::map( .x=.$data,
                            .f=~sim_dummy(  CqList=pluck(.$DNA, 1),
EPCR=.$EPCR, zeroAmount=.$zeroAmount,
                                            K=param.K, # variable
                                            beta=.$beta, diploid=.$diploid,
                                            maxtime=600, print.level=1, #600
                                            aux=c(  replicate=.$replicate,
rseed=.$rseed,
                                                    P=.$P, K=.$K,
                                                    ntrap=.$ntrap,
npertrap=.$npertrap, beta=.$beta  )  ) ),
                file=fout, append=TRUE, type=c("output") )
            cat(paste("Re-calculation for timeover ended in   ", Sys.time
(), "\n", sep=" "));
        }, silent=FALSE )
        print(proc.time()-ptime0); flush.console();
        temp[over.LID] <- temp.over

        try( {
            close(fout)
```

```
            cat(paste("The log object 'fout' was closed successfully.",
"\n", sep=""))
        } )
    }

    result <- om.each %>%
        dplyr::mutate(res=temp) %>%
        tidyr::hoist(.col=res, Time.obj="cal.time", obj="obj",
report="report") %>%
        dplyr::group_by(LID) %>%
        dplyr::mutate(  Time=pluck(Time.obj, 1, 3), Iter=pluck(obj, 1,
"iterations", 1)  ) %>%
        dplyr::mutate(
                        P_lwr=pluck(report, 1)[1, 5], P_est=pluck(report, 1)
[1, 1], P_upr=pluck(report, 1)[1, 6],
                        K_lwr=pluck(report, 1)[2, 5], K_est=pluck(report, 1)
[2, 1], K_upr=pluck(report, 1)[2, 6],
                        T_lwr=pluck(report, 1)[3, 5], T_est=pluck(report, 1)
[3, 1], T_upr=pluck(report, 1)[3, 6],
                        C_lwr=pluck(report, 1)[4, 5], C_est=pluck(report, 1)
[4, 1], C_upr=pluck(report, 1)[4, 6]
        ) %>%
        dplyr::select(-Time.obj, -obj, -report) %>%
        dplyr::ungroup()
    rm(temp)
    cat("Iteration succeeded.\n\n")
    rm(e1); gc(); gc();

    result <- result %>% arrange(LID)
    result.df <- select(result, -DNA, -starts_with("res"))
    print(summary(result.df)) # $DNA is a list; summary() got weird.
    assign(paste("r", analysis_name, session, iter.num, sep="."),
value=result)
    save(result, file=paste("r", analysis_name, session, iter.num, "rda",
sep="."))
    readr::write_csv(result.df, paste("r", analysis_name, session,
iter.num, "csv", sep="."))
    cat(paste("Writing", paste("r", analysis_name, session, iter.num, "csv",
sep="."), "completed.\n", sep=" "))
    flush.console()
    rm(result, result.df)
}
```

Estimation with `freqpcr()` with Core i9-7920X

- Beta , K=1 : 170 sec / full parameter region x 5 replicates (3120 rows) / 16 threads

- Beta , K=NULL: 1200 sec / full parameter region x 5 replicates (3120 rows) / 16 threads
- (16.5 h / 50 iters)
- Gamma, K=1 : 1500 sec / full parameter region x 5 replicates (3120 rows) / 16 threads
- (16.5 h / 50 iters)
- Gamma, K=NULL: 3500 sec / full parameter region x 5 replicates (3120 rows) / 16 threads
- Gamma, K=NULL: 40000 sec / full parameter region x 5 replicates (3120 rows) / 1 thread

2. Beta distribution, all parameters unknown

```
session <- "Beta_all" # variable
param.beta <- TRUE # variable
param.K <- NULL # variable

for (i in iter) {

    (replicate the code of the session "Beta_all")

}
```

3. Gamma distribution, K = 1

```
session <- "Gamma_K1" # variable
param.beta <- FALSE # variable
param.K <- 1 # variable

for (i in iter) {

    (replicate the code of the session "Beta_all")

}
```

4. Gamma distribution, all parameters unknown

```
session <- "Gamma_all" # variable
param.beta <- FALSE # variable
param.K <- NULL # variable

for (i in iter) {

    (replicate the code of the session "Beta_all")

}
```

Part IV Figures 4-

First, run the code of Part III. It takes a few MONTHS, depending on your environment. The summary of the calculation results are written to a csv file for each chunk.

Data post-processing of the numerical simulation

The following code chunks combine the result objects on an existing R session. Run after the numerical simulation.

```
library(tidyverse)
trialid <- 172308L
analysis_name <- paste("200824", trialid, sep=".")

# Beta distribution model: 200 replicates (both for "K1" and "all parameters
unknown")
iter <- 1:200
iter_ab <- paste(   formatC( as.integer(min(iter)), width=4, flag="0" ),
                    formatC( as.integer(max(iter)), width=4, flag="0" ),
sep="_"    )
sessions <- c("Beta_K1", "Beta_all")
for (k in sessions) {
    objects <- paste("r", analysis_name, k, formatC( as.integer(iter),
width=4, flag="0" ), sep=".")
    try( {
        for (j in 1:length(iter)) {
            assign(objects[j], readr::read_csv(paste(objects[j], "csv",
sep="."),

col_types="idddildddddddiddddlcdidddddddddddd"), envir=.GlobalEnv)
            print(get(objects[j]))
        }
```

```
        bind <- dplyr::bind_rows(mget(objects))
        assign(paste("r", analysis_name, k, iter_ab, sep="."), bind)
        save(bind, file=paste("r", analysis_name, k, iter_ab, "rda",
sep=".")) # 60 MB of the R binary file.
        readr::write_csv(bind, paste("r", analysis_name, k, iter_ab, "csv",
sep=".")) # 210 MB of the combined csv file.
    } )
}

# Gamma distribution model: first 50 replicates (both for "K1" and "all
parameters unknown")
iter <- 1:50
iter_ab <- paste(   formatC( as.integer(min(iter)), width=4, flag="0" ),
                    formatC( as.integer(max(iter)), width=4, flag="0" ),
sep="_"    )
sessions <- c("Gamma_all", "Gamma_K1")
for (k in sessions) {
    objects <- paste("r", analysis_name, k, formatC( as.integer(iter),
width=4, flag="0" ), sep=".")
    try( {
        for (j in 1:length(iter)) {
            assign(objects[j], readr::read_csv(paste(objects[j], "csv",
sep="."),

col_types="idddildddddddidddlcdidddddddddddd"), envir=.GlobalEnv)
            print(get(objects[j]))
        }
        bind <- dplyr::bind_rows(mget(objects))
        assign(paste("r", analysis_name, k, iter_ab, sep="."), bind)
        save(bind, file=paste("r", analysis_name, k, iter_ab, "rda",
sep=".")) # 15 MB of the R binary file.
        readr::write_csv(bind, paste("r", analysis_name, k, iter_ab, "csv",
sep=".")) # 50 MB of the combined csv file.
    } )
}

ls()
get(paste("r", analysis_name, sessions[1], iter_ab, sep="."))
```

Instead, you can download the combined results from figshare.com (https://figshare.com/collections/freqpcr/5258027).

The files are available as R binary files (saved using the code above).

Load the combined R binary files available from figshare.com

```
library(tidyverse)
trialid <- 172308L
analysis_name <- paste("200824", trialid, sep=".")
load(paste("r", analysis_name, "Beta_K1", "0001_0200", "rda", sep="."))
bind.Beta_K1 <- bind
load(paste("r", analysis_name, "Beta_all", "0001_0200", "rda", sep="."))
bind.Beta_all <- bind
load(paste("r", analysis_name, "Gamma_K1", "0001_0050", "rda", sep="."))
bind.Gamma_K1 <- bind
load(paste("r", analysis_name, "Gamma_all", "0001_0050", "rda", sep="."))
bind.Gamma_all <- bind
rm(bind)
ls()
```

The combined data seem like:

```
> bind.Beta_K1
# A tibble: 624,000 x 34
     LID     P     K ntrap npertrap replicate diploid scaleDNA targetScale
baseChange  EPCR zeroAmount sdMeasure ntotal  rseed
   <int> <dbl> <dbl> <dbl>    <dbl>     <int> <lgl>      <dbl>       <dbl>
<dbl> <dbl>      <dbl>     <dbl>  <dbl>  <int>
 1     1  0.01     1     2        2         1 FALSE    0.000001
1.2        0.24  0.97     0.0016       0.2      4 365190
 2     2  0.01     1     2        2         2 FALSE    0.000001
1.2        0.24  0.97     0.0016       0.2      4 272934
 3     3  0.01     1     2        2         3 FALSE    0.000001
1.2        0.24  0.97     0.0016       0.2      4 357485
 4     4  0.01     1     2        2         4 FALSE    0.000001
1.2        0.24  0.97     0.0016       0.2      4 220405
 5     5  0.01     1     2        2         5 FALSE    0.000001
1.2        0.24  0.97     0.0016       0.2      4 537430
 6  1001  0.01     1     2        4         1 FALSE    0.000001
1.2        0.24  0.97     0.0016       0.2      8 460547
 7  1002  0.01     1     2        4         2 FALSE    0.000001
1.2        0.24  0.97     0.0016       0.2      8  33196
 8  1003  0.01     1     2        4         3 FALSE    0.000001
1.2        0.24  0.97     0.0016       0.2      8 546004
 9  1004  0.01     1     2        4         4 FALSE    0.000001
```

```
10  1005  0.01     1     2        4         5 FALSE    0.000001
1.2       0.24  0.97     0.0016      0.2      8 434526
# ... with 623,990 more rows, and 19 more variables: ave_P_mean <dbl>,
ave_P_sd <dbl>, ave_P_est <dbl>, beta <lgl>,
#   Session <chr>, Time <dbl>, Iter <int>, P_lwr <dbl>, P_est <dbl>, P_upr
<dbl>, K_lwr <dbl>, K_est <dbl>, K_upr <dbl>,
#   T_lwr <dbl>, T_est <dbl>, T_upr <dbl>, C_lwr <dbl>, C_est <dbl>, C_upr
<dbl>
```

code for Figure 4 and after

Figure 4 is the estimation accuracy of p

```
print(analysis_name)

library(magrittr)
library(extrafont)
library(ggplot2)
library(ggthemes)
library(cowplot)
library(devEMF)

caption <- "est_P"
sessions <- c("Beta_all", "Beta_K1", "Gamma_all", "Gamma_K1")
sessions.label <- c(Beta_all="freqpcr: Beta distribution, all parameters
unknown",
                    Beta_K1="freqpcr: Beta distribution, k was fixed 1",
                    Gamma_all="freqpcr: Gamma distribution, all parameters
unknown",
                    Gamma_K1="freqpcr: Gamma distribution, k was fixed 1")
for (k in sessions) {
    dat.bind <- paste("bind", k, sep=".") %>% get()
    dat.nest <- dat.bind %>%
        dplyr::arrange(LID) %>%
        dplyr::group_by(P, K, ntrap, npertrap, ntotal, diploid) %>%
        tidyr::nest() %>%
        dplyr::mutate(  zeroci=binom.test(0, as.integer(ntotal))$conf.int
[2]  ) %>%
        dplyr::mutate(  used.binom=P>=zeroci,
                        used.three=ntotal>=3.0/P  )
    print(dat.bind)
    print(dat.nest)
    levels_p <- tibble(P=c(0.01, 0.05, 0.1, 0.25, 0.5, 0.75), level=c(0.01,
0.05, 0.1, 0.25, 0.5, 0.75))
```

```
    THREE <- c(hsv(0.4, 0, 0.8), hsv(0.4, 0, 1)) # color chart: is the
sample size enough
    p <-ggplot(data=dat.bind) +
        geom_rect(  data=dat.nest, aes(xmin=0, xmax=Inf, ymin=0, ymax=1,
fill=used.three), color=NA ) +
        geom_hline( yintercept=c(0, 0.5, 1), linetype=1, size=0.25 ) +
        geom_hline( data=levels_p, aes(yintercept=level), linetype="dotted",
color="black", size=0.25 ) +
        geom_boxplot(   aes(y=P_upr, x=ntrap, group=npertrap), notch=FALSE,
                        color=hsv(0.18, 0.99, 0.1), fill=hsv(0.18, 0.99,
0.99), outlier.shape=NA, size=0.25,
                        width=1.7/length(unique(dat.bind$npertrap)),
fatten=1.5   ) + # fatten: width of median
        geom_boxplot(   aes(y=P_lwr, x=ntrap, group=npertrap), notch=FALSE,
                        color=hsv(0.58, 0.99, 0.25), fill=hsv(0.53, 0.9,
0.99), outlier.shape=NA, size=0.2,
                        width=1.5/length(unique(dat.bind$npertrap)),
fatten=1.5   ) +
        geom_boxplot(   aes(y=P_est, x=ntrap, group=npertrap), notch=FALSE,
                        color=hsv(0.5, 0, 0.0), fill=hsv(0.5, 0, 1),
outlier.shape=NA, size=0.2,
                        width=1.0/length(unique(dat.bind$npertrap)),
fatten=1.5   ) +
        facet_grid(P ~ ntotal,  margins=F, labeller=purrr::partial
(label_both, sep=" = ")) +
        theme_classic(base_size=9) +
        scale_fill_manual(values=THREE) +
        theme(  panel.background=element_blank(),
plot.background=element_rect(colour=NA, fill=NA),
                plot.margin=unit(c(0.25, 0, 0.25, 0.25), "lines")  ) + #
top, right, bottom, left
        theme(  text=element_text(family="Arial"),# plot.title=element_blank
(),
                plot.title=element_text(family="Arial", face="bold", size=8,
angle=0, hjust=0),
                strip.background=element_blank(),
                strip.text=element_text(family="Arial Narrow", face="bold",
size=8,
                                        margin=margin(b=2, t=1, l=4, r=4,
unit="pt")),
                axis.text.x=element_text(family="Arial Narrow", size=6,
angle=0, hjust=0.5),
                axis.text.y=element_text(family="Arial Narrow", size=6,
angle=0, hjust=1)  ) +
        theme(  axis.line=element_line(size=0.3, color="black"),
                axis.ticks=element_line(size=0.3, color="black") ,
                axis.ticks.length=unit(0.5, "mm")  ) +
        theme(legend.position="none") +
```

```
        scale_x_log10( breaks=c(1, 2, 4, 8, 16, 32, 64, 128), labels=c(1,
2, 4, 8, 16, 32, 64, 128)) +
        scale_y_continuous( limit=c(0, 1), breaks=c(0, 0.25, 0.5, 0.75, 1),
labels=c("0", "", "0.5", "", "1")) +
        labs(   title=sessions.label[k], x="Number of bulk DNA samples
(ntrap)",
                y="Estimated p (95% CI Lower | MLE | 95% CI Upper)")
    outer.title <- paste("Fig", caption, k, sep=".")
    assign(paste("ggp", outer.title, sep="."), value=p)
    ggsave(paste(outer.title, ".pdf", sep=""), device=cairo_pdf, plot=p,
dpi=300, width=11/2.54, height=9.6/2.54)
    ggsave(paste(outer.title, ".eps", sep=""), device=cairo_ps, plot=p,
dpi=300, width=11/2.54, height=9.6/2.54)
    ggsave(paste(outer.title, ".png", sep=""), type="cairo", plot=p,
dpi=300, width=11/2.54, height=9.6/2.54)
    # save as the enhanced metafile
    emf(file=paste(outer.title, ".emf", sep=""), width=11/2.54,
height=9.6/2.54)
    print(p)
    dev.off()
}
warnings()

# combine pdf files
library(pdftools)
pdf_combine(paste("Fig", caption, sessions, "pdf", sep="."), output=paste
("Fig", caption, "combined", "pdf", sep="."))

# Figure files for submission
file.copy(from="Fig.est_P.Beta_all.eps", to="Figure4.eps", overwrite=TRUE,
copy.date=TRUE)
file.copy(from="Fig.est_P.Beta_all.pdf", to="Figure4.pdf", overwrite=TRUE,
copy.date=TRUE)
file.copy(from="Fig.est_P.Beta_all.emf", to="Figure4.emf", overwrite=TRUE,
copy.date=TRUE)

file.copy(from="Fig.est_P.Beta_K1.eps", to="FigureS3.eps", overwrite=TRUE,
copy.date=TRUE)
file.copy(from="Fig.est_P.Beta_K1.pdf", to="FigureS3.pdf", overwrite=TRUE,
copy.date=TRUE)
file.copy(from="Fig.est_P.Beta_K1.emf", to="FigureS3.emf", overwrite=TRUE,
copy.date=TRUE)

file.copy(from="Fig.est_P.Gamma_all.eps", to="FigureS4.eps", overwrite=TRUE,
copy.date=TRUE)
file.copy(from="Fig.est_P.Gamma_all.pdf", to="FigureS4.pdf", overwrite=TRUE,
copy.date=TRUE)
```

```
file.copy(from="Fig.est_P.Gamma_all.emf", to="FigureS4.emf", overwrite=TRUE,
copy.date=TRUE)
```

Estimation of P by simple averaging of ΔΔCq values (Figure 5)

```
caption <- "est_averaging"
sessions <- c("Beta_all")
sessions.label <- c(Beta_all="Estimation by averaging \u0394\u0394Cq
values")

for (k in sessions) {
    dat.bind <- paste("bind", k, sep=".") %>% get() %>%
        dplyr::mutate(  ave_P_se=ave_P_sd/sqrt(ntrap),
                        ave_P_est_recalc=(1+EPCR)^(-ave_P_mean),
                        ave_P_lwr=(1+EPCR)^(-ave_P_mean-1.96*ave_P_se),
                        ave_P_upr=(1+EPCR)^(-ave_P_mean+1.96*ave_P_se)  )
    dat.nest <- dat.bind %>%
        dplyr::arrange(LID) %>%
        dplyr::group_by(P, K, ntrap, npertrap, ntotal, diploid) %>%
        tidyr::nest() %>%
        dplyr::mutate(  zeroci=binom.test(0, as.integer(ntotal))$conf.int
[2]  ) %>%
        dplyr::mutate(  used.binom=P>=zeroci,
                        used.three=ntotal>=3.0/P  )
    levels_p <- tibble(P=c(0.01, 0.05, 0.1, 0.25, 0.5, 0.75), level=c(0.01,
0.05, 0.1, 0.25, 0.5, 0.75))
    THREE <- c(hsv(0.4, 0, 0.8), hsv(0.4, 0, 1)) # color chart if the sample
size is enough

    p <-ggplot(dat.bind) +
        geom_rect(  data=dat.nest, aes(xmin=0, xmax=Inf, ymin=0, ymax=1,
fill=used.three), color=NA ) +
        geom_hline( yintercept=c(0, 0.5, 1), linetype=1, size=0.25 ) +
        geom_hline( data=levels_p, aes(yintercept=level), linetype="dotted",
color="black", size=0.25 ) +
        geom_boxplot(   aes(y=ave_P_upr, x=ntrap, group=npertrap),
notch=FALSE,
                        color=hsv(0.18, 0.99, 0.1), fill=hsv(0.18, 0.99,
0.99), outlier.shape=NA, size=0.25,
                        width=1.7/length(unique(dat.bind$npertrap)),
fatten=1.5   ) + # fatten: width of median
        geom_boxplot(   aes(y=ave_P_lwr, x=ntrap, group=npertrap),
notch=FALSE,
```

```
                        color=hsv(0.58, 0.99, 0.25), fill=hsv(0.53, 0.9,
0.99), outlier.shape=NA, size=0.2,
                        width=1.5/length(unique(dat.bind$npertrap)),
fatten=1.5   ) +
        geom_boxplot(   aes(y=ave_P_est, x=ntrap, group=npertrap),
notch=FALSE,
                        color=hsv(0.5, 0, 0.0), fill=hsv(0.5, 0, 1),
outlier.shape=NA, size=0.2,
                        width=1.0/length(unique(dat.bind$npertrap)),
fatten=1.5   ) +
        facet_grid(P ~ ntotal,  margins=F, labeller=purrr::partial
(label_both, sep=" = ")) +
        theme_classic(base_size=9) +
        scale_fill_manual(values=THREE) +
        theme(  panel.background=element_blank(),
plot.background=element_rect(colour=NA, fill=NA),
                plot.margin=unit(c(0.25, 0, 0.25, 0.25), "lines")  ) + #
top, right, bottom, left
        theme(  text=element_text(family="Arial"),# plot.title=element_blank
(),
                plot.title=element_text(family="Arial", face="bold", size=8,
angle=0, hjust=0),
                strip.background=element_blank(),
                strip.text=element_text(family="Arial Narrow", face="bold",
size=8,
                                        margin=margin(b=2, t=1, l=4, r=4,
unit="pt")),
                axis.text.x=element_text(family="Arial Narrow", size=6,
angle=0, hjust=0.5),
                axis.text.y=element_text(family="Arial Narrow", size=6,
angle=0, hjust=1)  ) +
        theme(  axis.line=element_line(size=0.3, color="black"),
                axis.ticks=element_line(size=0.3, color="black") ,
                axis.ticks.length=unit(0.5, "mm")  ) +
        theme(legend.position="none") +
        scale_x_log10( breaks=c(1, 2, 4, 8, 16, 32, 64, 128), labels=c(1,
2, 4, 8, 16, 32, 64, 128)) +
        scale_y_continuous( limit=c(0, 1.5), breaks=c(0, 0.25, 0.5, 0.75,
1, 1.25, 1.5),
                            labels=c("0", "", "0.5", "", "1", "", "") ) +
        labs(   title=sessions.label[k], x="Number of bulk DNA samples
(ntrap)",
                y="Estimated p (Mean - 1.96 SE | Mean | Mean + 1.96 SE)"   )
    outer.title <- paste("Fig", caption, sep=".")
    assign(paste("ggp", outer.title, sep="."), value=p)
    ggsave(paste(outer.title, ".pdf", sep=""), device=cairo_pdf, plot=p,
dpi=300, width=11/2.54, height=9.6/2.54)
    ggsave(paste(outer.title, ".eps", sep=""), device=cairo_ps, plot=p,
```

```
dpi=300, width=11/2.54, height=9.6/2.54)
    ggsave(paste(outer.title, ".png", sep=""), type="cairo", plot=p,
dpi=300, width=11/2.54, height=9.6/2.54)
    # save as the enhanced metafile
    emf(file=paste(outer.title, ".emf", sep=""), width=11/2.54,
height=9.6/2.54)
    print(p)
    dev.off()
}
warnings()

# Figure files for submission
file.copy(from="Fig.est_averaging.eps", to="Figure5.eps", overwrite=TRUE,
copy.date=TRUE)
file.copy(from="Fig.est_averaging.pdf", to="Figure5.pdf", overwrite=TRUE,
copy.date=TRUE)
file.copy(from="Fig.est_averaging.emf", to="Figure5.emf", overwrite=TRUE,
copy.date=TRUE)
```

Figures S5--S7: Time/iterations to converge with fitqpcr()

```
caption <- c("time", "iters")
sessions <- c("Beta_all", "Beta_K1", "Gamma_all", "Gamma_K1")
sessions.label <- c(Beta_all="freqpcr: Beta distribution, all parameters
unknown",
                    Beta_K1="freqpcr: Beta distribution, k was fixed 1",
                    Gamma_all="freqpcr: Gamma distribution, all parameters
unknown",
                    Gamma_K1="freqpcr: Gamma distribution, k was fixed 1")
for (k in sessions) {
    dat.bind <- paste("bind", k, sep=".") %>% get()
    dat.nest <- dat.bind %>%
        dplyr::arrange(LID) %>%
        dplyr::group_by(P, K, ntrap, npertrap, ntotal, diploid) %>%
        tidyr::nest() %>%
        dplyr::mutate(  zeroci=binom.test(0, as.integer(ntotal))$conf.int
[2]  ) %>%
        dplyr::mutate(  used.binom=P>=zeroci,
                        used.three=ntotal>=3.0/P  )
#    levels_p <- tibble(P=c(0.01, 0.05, 0.1, 0.25, 0.5, 0.75), level=c(0.01,
0.05, 0.1, 0.25, 0.5, 0.75))
    THREE <- c(hsv(0.4, 0, 0.8), hsv(0.4, 0, 1)) # color chart if the sample
size is enough
```

```
    p <-ggplot(dat.bind) +
        geom_rect(  data=dat.nest,
                    aes(xmin=0, xmax=Inf, ymin=0, ymax=Inf,
fill=used.three), color=NA ) +
        geom_hline( yintercept=c(seq(0.1, 1, by=0.1), seq(1, 10, by=1), seq
(10, 60, by=10), seq(60, 600, by=60)),
                    linetype=1, color=gray(0.88), size=0.05) +
        geom_hline( yintercept=c(1, 60, 3600), linetype=1, size=0.25 ) +
        geom_hline( yintercept=c(0.1, 10, 600), linetype=3, size=0.25 ) +
        geom_boxplot(   aes(y=Time, x=ntrap, group=npertrap), notch=FALSE,
                        color=hsv(0.5, 0, 0.15), fill=hsv(0.5, 0, 1, 0.9),
size=0.25,
                        outlier.shape=NA, outlier.size=0.05,
outlier.color=gray(0.2),
                        width=1.6/length(unique(dat.bind$npertrap)),
fatten=1.0   ) +
        facet_grid(P ~ ntotal,  margins=F, labeller=purrr::partial
(label_both, sep=" = ")) +
        theme_classic(base_size=9) +
        scale_fill_manual(values=THREE) +
        theme(  panel.background=element_blank(),
plot.background=element_rect(colour=NA, fill=NA),
                plot.margin=unit(c(0.25, 0, 0.25, 0.25), "lines")  ) + #
top, right, bottom, left
        theme(  text=element_text(family="Arial"),
                plot.title=element_text(family="Arial", face="bold", size=8,
angle=0, hjust=0),
                strip.background=element_blank(),
                strip.text=element_text(family="Arial Narrow", face="bold",
size=8, margin=margin(b=2, t=1, l=4, r=4, unit="pt")),
                axis.text.x=element_text(family="Arial Narrow", size=6,
angle=0, hjust=0.5),
                axis.text.y=element_text(family="Arial Narrow", size=6,
angle=0, hjust=1)  ) +
        theme(legend.position="none") +
        scale_x_log10( breaks=c(1, 2, 4, 8, 16, 32, 64, 128), labels=c(1,
2, 4, 8, 16, 32, 64, 128)) +
        scale_y_log10( limit=c(0.01, 1200), breaks=c(0.1, 1, 10, 60, 600,
3600),
                                        labels=c("0.1 s", "1 s", "10 s", "1
m", "10 m", "1 h")) +
        labs(title=sessions.label[k], x="Number of bulk DNA samples
(ntrap)", y="Time\n") #
    outer.title <- paste("Fig", caption[1], k, sep=".")
    assign(paste("ggp", outer.title, sep="."), value=p)

    p <-ggplot(dat.bind) +
        geom_rect(  data=filter(dat.nest),
```

```
                        aes(xmin=0, xmax=Inf, ymin=-Inf, ymax=Inf,
fill=used.three), color=NA ) +
        geom_hline( yintercept=seq(0, 100, by=10), linetype=1, color=gray
(0.88), size=0.1) +
        geom_hline(yintercept=c(1, 50, 100), linetype=1, size=0.25) +
        geom_boxplot(   aes(y=Iter, x=ntrap, group=npertrap), notch=FALSE,
                        color=hsv(0.5, 0, 0.15), fill=hsv(0.5, 0, 1, 0.9),
size=0.25,
                        outlier.shape=NA, outlier.size=0.05,
outlier.color=gray(0.2),
                        width=1.6/length(unique(dat.bind$npertrap)),
fatten=1.0   ) +
        facet_grid(P ~ ntotal,  margins=F, labeller=purrr::partial
(label_both, sep=" = ")) +
        theme_classic(base_size=9) +
        theme(aspect.ratio=1) + # keep the aspect ratio regardless of
margin.
        scale_fill_manual(values=THREE) +
        theme(  panel.background=element_blank(),
plot.background=element_rect(colour=NA, fill=NA),
                plot.margin=unit(c(0.25, 0, 0.25, 0.25), "lines")  ) + #
top, right, bottom, left
        theme(  text=element_text(family="Arial"),
                plot.title=element_text(family="Arial", face="bold", size=8,
angle=0, hjust=0),
                strip.background=element_blank(),
                strip.text=element_text(family="Arial Narrow", face="bold",
size=8, margin=margin(b=2, t=1, l=4, r=4, unit="pt")),
                axis.text.x=element_text(family="Arial Narrow", size=6,
angle=0, hjust=0.5),
                axis.text.y=element_text(family="Arial Narrow", size=6,
angle=0, hjust=1)  ) +
        theme(legend.position="none") +
        scale_x_log10( breaks=c(1, 2, 4, 8, 16, 32, 64, 128), labels=c(1,
2, 4, 8, 16, 32, 64, 128)) +
        scale_y_continuous( limit=c(0, 100), breaks=c(0, 50, 100)) +
        labs(title=sessions.label[k], x="Number of bulk DNA samples
(ntrap)", y="Iterations\n") #\n
    outer.title <- paste("Fig", caption[2], k, sep=".")
    assign(paste("ggp", outer.title, sep="."), value=p)

    # Making conbined figure via cowplot
    library(cowplot)
    ps2 <- cowplot::plot_grid(  get(paste("ggp.Fig.time", k, sep=".")),
                                get(paste("ggp.Fig.iters", k, sep=".")),
                                align="v", rel_heights=c(1, 1), scale=c(1,
1), ncol=1,
                                labels=c("A", "B"), label_size=12,
```

```
hjust=0.05  )
    outer.title <- paste("Fig", paste(caption, collapse="."), k, sep=".")
    ggsave(paste(outer.title, ".eps", sep=""), device=cairo_ps, plot=ps2,
dpi=300, width=11/2.54, height=22.8/2.54)
    ggsave(paste(outer.title, ".pdf", sep=""), device=cairo_pdf, plot=ps2,
dpi=300, width=11/2.54, height=22.8/2.54)
    emf(file=paste(outer.title, ".emf", sep=""), width=11/2.54,
height=22.8/2.54)
    print(ps2)
    dev.off()

}
warnings()

# combine pdf files
library(pdftools)
pdf_combine(paste("Fig", paste(caption, collapse="."), sessions, "pdf",
sep="."),
            output=paste("Fig", paste(caption, collapse="."), "combined",
"pdf", sep="."))

# Figure files for submission
file.copy(from="Fig.time.iters.Beta_all.eps", to="FigureS5.eps",
overwrite=TRUE, copy.date=TRUE)
file.copy(from="Fig.time.iters.Beta_all.pdf", to="FigureS5.pdf",
overwrite=TRUE, copy.date=TRUE)
file.copy(from="Fig.time.iters.Beta_all.emf", to="FigureS5.emf",
overwrite=TRUE, copy.date=TRUE)
file.copy(from="Fig.time.iters.Beta_K1.eps", to="FigureS6.eps",
overwrite=TRUE, copy.date=TRUE)
file.copy(from="Fig.time.iters.Beta_K1.pdf", to="FigureS6.pdf",
overwrite=TRUE, copy.date=TRUE)
file.copy(from="Fig.time.iters.Beta_K1.emf", to="FigureS6.emf",
overwrite=TRUE, copy.date=TRUE)
file.copy(from="Fig.time.iters.Gamma_all.eps", to="FigureS7.eps",
overwrite=TRUE, copy.date=TRUE)
file.copy(from="Fig.time.iters.Gamma_all.pdf", to="FigureS7.pdf",
overwrite=TRUE, copy.date=TRUE)
file.copy(from="Fig.time.iters.Gamma_all.emf", to="FigureS7.emf",
overwrite=TRUE, copy.date=TRUE)
```

Success probability of the simultaneous parameter estimation

```
> bind.Beta_all
# A tibble: 624,000 x 34
     LID     P     K ntrap npertrap replicate diploid scaleDNA targetScale
baseChange  EPCR zeroAmount sdMeasure ntotal  rseed
   <int> <dbl> <dbl> <dbl>    <dbl>     <int> <lgl>      <dbl>       <dbl>
<dbl> <dbl>      <dbl>     <dbl>  <dbl>  <int>
 1     1  0.01     1     2        2         1 FALSE    0.000001
1.2        0.24  0.97     0.0016       0.2      4 365190
 2     2  0.01     1     2        2         2 FALSE    0.000001
1.2        0.24  0.97     0.0016       0.2      4 272934
 3     3  0.01     1     2        2         3 FALSE    0.000001
1.2        0.24  0.97     0.0016       0.2      4 357485
 4     4  0.01     1     2        2         4 FALSE    0.000001
1.2        0.24  0.97     0.0016       0.2      4 220405
 5     5  0.01     1     2        2         5 FALSE    0.000001
1.2        0.24  0.97     0.0016       0.2      4 537430
 6  1001  0.01     1     2        4         1 FALSE    0.000001
1.2        0.24  0.97     0.0016       0.2      8 460547
 7  1002  0.01     1     2        4         2 FALSE    0.000001
1.2        0.24  0.97     0.0016       0.2      8  33196
 8  1003  0.01     1     2        4         3 FALSE    0.000001
1.2        0.24  0.97     0.0016       0.2      8 546004
 9  1004  0.01     1     2        4         4 FALSE    0.000001
1.2        0.24  0.97     0.0016       0.2      8 552619
10  1005  0.01     1     2        4         5 FALSE    0.000001
1.2        0.24  0.97     0.0016       0.2      8 434526
# ... with 623,990 more rows, and 19 more variables: ave_P_mean <dbl>,
ave_P_sd <dbl>, ave_P_est <dbl>, beta <lgl>,
#   Session <chr>, Time <dbl>, Iter <int>, P_lwr <dbl>, P_est <dbl>, P_upr
<dbl>, K_lwr <dbl>, K_est <dbl>, K_upr <dbl>,
#   T_lwr <dbl>, T_est <dbl>, T_upr <dbl>, C_lwr <dbl>, C_est <dbl>, C_upr
<dbl>
```

Example code to calculate success probabilities. No need to run here.

```
Beta_all.nest <- bind.Beta_all %>%
    dplyr::arrange(LID) %>%
    dplyr::group_by(P, K, ntrap, npertrap, ntotal, diploid) %>%
    tidyr::nest() %>%
```

```
    dplyr::mutate(  zeroci=binom.test(0, as.integer(ntotal))$conf.int[2]  )
%>%
    dplyr::mutate(  used.binom=P>=zeroci,
                    used.three=ntotal>=3.0/P,
                    ns.P_lwr=purrr::map_dbl(.x=data, .f=~{sum(!is.na
(.$P_lwr))/length(.$P_lwr)}),
                    ns.K_lwr=purrr::map_dbl(.x=data, .f=~{sum(!is.na
(.$K_lwr))/length(.$K_lwr)}),
                    ns.T_lwr=purrr::map_dbl(.x=data, .f=~{sum(!is.na
(.$T_lwr))/length(.$T_lwr)}),
                    ns.C_lwr=purrr::map_dbl(.x=data, .f=~{sum(!is.na
(.$C_lwr))/length(.$C_lwr)})  )
Beta_all.nest
summary(select(Beta_all.nest, -data)) # Mean is the success probability
summary(select(filter(Beta_all.nest, used.three), -data)) # The success
probability when ntotal satisfies 3/P
```

```
> summary(select(Beta_all.nest, -data)) # Mean is the success probability
       P                 K            ntrap        npertrap        diploid
ntotal            zeroci
 Min.   :0.0100   Min.   : 1.0   Min.   : 2   Min.   : 1.000   Mode :logical
Min.   :  4.00   Min.   :0.02841
 1st Qu.:0.0500   1st Qu.: 2.5   1st Qu.: 4   1st Qu.: 2.000
FALSE:624        1st Qu.: 16.00   1st Qu.:0.05601
 Median :0.1750   Median : 6.0   Median : 8   Median :
4.000                  Median : 32.00   Median :0.10888
 Mean   :0.2767   Mean   :10.0   Mean   :14   Mean   :
9.423                  Mean   : 54.15   Mean   :0.16106
 3rd Qu.:0.5000   3rd Qu.:13.5   3rd Qu.:16   3rd Qu.:
8.000                  3rd Qu.: 64.00   3rd Qu.:0.20591
 Max.   :0.7500   Max.   :27.0   Max.   :64   Max.
:64.000                  Max.   :128.00   Max.   :0.60236
 used.binom       used.three         ns.P_lwr          ns.K_lwr
ns.T_lwr          ns.C_lwr
 Mode :logical   Mode :logical   Min.   :0.0000   Min.   :0.0000   Min.
:0.0000   Min.   :0.0000
 FALSE:268       FALSE:224       1st Qu.:0.4407   1st Qu.:0.2767   1st
Qu.:0.4273   1st Qu.:0.4355
 TRUE :356       TRUE :400       Median :0.9730   Median :0.7600
Median :0.8725   Median :0.9340
                                 Mean   :0.7061   Mean   :0.5988
Mean   :0.6806   Mean   :0.6964
                                 3rd Qu.:0.9990   3rd Qu.:0.8745   3rd
Qu.:0.9790   3rd Qu.:0.9840
```

```
                                Max.   :1.0000   Max.   :0.9980   Max.   
:1.0000   Max.   :1.0000   
```

```
> summary(select(filter(Beta_all.nest, used.three), -data)) # The success
probability when ntotal satisfies 3/P
       P                K             ntrap            npertrap      diploid       
ntotal           zeroci       
 Min.   :0.0500   Min.   : 1.0   Min.   : 2.00   Min.   : 1.00   
Mode :logical   Min.   :  4.00   Min.   :0.02841  
 1st Qu.:0.1000   1st Qu.: 2.5   1st Qu.: 4.00   1st Qu.: 2.00   
FALSE:400       1st Qu.: 32.00   1st Qu.:0.02841  
 Median :0.3750   Median : 6.0   Median : 8.00   Median : 
4.00                   Median : 64.00   Median :0.05601  
 Mean   :0.3905   Mean   :10.0   Mean   :16.32                   
Mean   :11.31                  Mean   : 66.48   Mean   :0.10602  
 3rd Qu.:0.7500   3rd Qu.:13.5   3rd Qu.:16.00   3rd 
Qu.:16.00                  3rd Qu.:128.00   3rd Qu.:0.10888  
 Max.   :0.7500   Max.   :27.0   Max.   :64.00   Max.   
:64.00                   Max.   :128.00   Max.   :0.60236  
 used.binom       used.three        ns.P_lwr          ns.K_lwr         
ns.T_lwr         ns.C_lwr     
 Mode :logical   Mode:logical   Min.   :0.0000   Min.   :0.0000   Min.   
:0.0000   Min.   :0.0000  
 FALSE:44        TRUE:400       1st Qu.:0.9760   1st Qu.:0.7758   1st 
Qu.:0.8882   1st Qu.:0.9400  
 TRUE :356                      Median :0.9965   Median :0.8460   
Median :0.9660   Median :0.9735  
                                Mean   :0.8434   Mean   :0.7413   
Mean   :0.8083   Mean   :0.8271  
                                3rd Qu.:1.0000   3rd Qu.:0.9313   3rd 
Qu.:0.9842   3rd Qu.:0.9890  
                                Max.   :1.0000   Max.   :0.9980   Max.   
:1.0000   Max.   :1.0000   
```

Figure S1, S2: estimation success

Succecc/fail of the estimation process for each parameter.

> Note: "success" only matters some result value has been obtained: the result may deviate from its true value.

```
caption <- "success"
sessions <- c("Beta_all", "Beta_K1", "Gamma_all", "Gamma_K1")
sessions.label <- c(Beta_all="freqpcr: Beta distribution, all parameters
unknown",
                    Beta_K1="freqpcr: Beta distribution, k was fixed 1",
                    Gamma_all="freqpcr: Gamma distribution, all parameters
unknown",
                    Gamma_K1="freqpcr: Gamma distribution, k was fixed 1")
i <- 1
for (k in sessions) {
    dat.bind <- paste("bind", k, sep=".") %>% get()
    dat.nest <- dat.bind %>%
        dplyr::arrange(LID) %>%
        dplyr::group_by(P, K, ntrap, npertrap, ntotal, diploid) %>%
        tidyr::nest() %>%
        dplyr::mutate(  zeroci=binom.test(0, as.integer(ntotal))$conf.int
[2]  ) %>%
        dplyr::mutate(  used.binom=P>=zeroci,
                        used.three=ntotal>=3.0/P,
                        ns.P_lwr=purrr::map_dbl(.x=data, .f=~{sum(!is.na
(.$P_lwr))/length(.$P_lwr)}),
                        ns.K_lwr=purrr::map_dbl(.x=data, .f=~{sum(!is.na
(.$K_lwr))/length(.$K_lwr)}),
                        ns.T_lwr=purrr::map_dbl(.x=data, .f=~{sum(!is.na
(.$T_lwr))/length(.$T_lwr)}),
                        ns.C_lwr=purrr::map_dbl(.x=data, .f=~{sum(!is.na
(.$C_lwr))/length(.$C_lwr)})  )
    dat.nest.longer <- dat.nest %>%
        tidyr::pivot_longer( cols=starts_with("ns."),
                             names_to=c("ns", "Parameter"), values_to=c
("pSuccess"), names_sep="\\." )

    data <- dat.nest.longer
    data$Parameter <- factor(data$Parameter, levels=c("P_lwr", "K_lwr",
"T_lwr", "C_lwr"))
    label_parameter <- c(   P_lwr=expression(italic(p)), K_lwr=expression
(italic(k)),
                            T_lwr=expression(delta[T]), C_lwr=expression
(epsilon[c])   )
    levels_p <- tibble(P=c(0.01, 0.05, 0.1, 0.25, 0.5, 0.75), level=c(0.01,
0.05, 0.1, 0.25, 0.5, 0.75))
    RFREQ <- c(hsv(0, 0, 0), hsv(0.27, 0.95, 0.75, i), hsv(0.95, 0.9, 0.9),
hsv(0.72, 0.74, 0.95), hsv(0.15, 0.99, 0.8))

    p <-ggplot(data, aes()) +
        geom_vline( xintercept=c(seq(0.01, 0.1, by=0.01), seq(0.1, 1,
by=0.1)),
```

```
                        linetype=1, color=gray(0.5), size=0.2) +
            geom_vline(xintercept=c(0.01, 0.1, 1), linetype=1, size=0.4) +
            geom_rect(  aes(xmin=0.01, xmax=1, ymin=0, ymax=1), fill=hsv(0.15,
0.01, 1), color="black", size=0.25) +
            geom_rect(  data=dat.nest, aes(xmin=0.01, xmax=3.0/ntotal, ymin=0,
ymax=1), color=NA, fill=gray(0.8) ) +
            geom_hline(yintercept=c(0, 0.5, 1), linetype=1, size=0.25) +
            geom_line(aes(y=pSuccess, x=P, group=interaction(K, Parameter),
color=Parameter, linetype=Parameter), size=0.4) +
            geom_point(aes(y=pSuccess, x=P, group=interaction(K, Parameter),
color=Parameter, shape=Parameter), size=1.05) +
            scale_color_manual(values=RFREQ, name="Parameter",
labels=label_parameter) +
            scale_linetype(name="Parameter", labels=label_parameter) +
            scale_shape(name="Parameter", labels=label_parameter) +
            facet_grid(ntrap + npertrap ~ K,  margins=F, labeller=purrr::partial
(label_both, sep=" = ")) +
            theme_classic(base_size=9) +
            theme(  panel.background=element_blank(),
plot.background=element_rect(colour=NA, fill=NA),
                    plot.margin=unit(c(0, 0, 0.25, 0.25), "lines")  ) + # top,
right, bottom, left
            theme(  text=element_text(family="Arial"),
                    plot.title=element_text(family="Arial", face="bold", size=8,
angle=0, hjust=0),
                    strip.background=element_blank(),
                    strip.text.x=element_text(  family="Arial Narrow",
face="bold", size=8,
                                                margin=margin(b=2, t=2, l=4,
r=0, unit="pt")  ),
                    strip.text.y=element_text(  family="Arial Narrow",
face="bold", size=8, angle=0, hjust=0  ),
                    axis.text.x=element_text(family="Arial Narrow", size=6,
angle=0, hjust=0.5),
                    axis.text.y=element_text(family="Arial Narrow", size=6,
angle=0, hjust=1)  ) +
            theme(  legend.position="top", legend.spacing=unit(0, "lines"),
                    legend.margin=margin(b=0, t=0, l=8, r=4, unit="pt"), # the
margin around each legend
                    legend.box="vertical",
                    legend.box.margin=margin(b=0, t=0, l=0, r=0, unit="pt"), #
margins around the full legend area
                    legend.box.background=element_blank(),
                    legend.box.spacing=unit(4, unit="pt"),
                    legend.key.size=unit(1.5, "lines"), legend.key.height=unit
(1, "pt"), # enlarge key but not increase margin
                    legend.title=element_text(family="Arial", size=8),
                    legend.text=element_text(family="Arial", size=8)  ) +
```

```
        scale_x_log10(  limit=c(0.01, 1), breaks=c(0.01, 0.1, 0.5, 1),
labels=c(0.01, 0.1, 0.5, 1)  ) +
        scale_y_continuous( expand=c(0.1, 0), limit=c(0, 1), breaks=c(0,
0.5, 1) ) +
        labs(title=sessions.label[k], x="True size of p (R allele
frequency)", y="Success probability of interval estimation")

    outer.title <- paste("Fig", caption, k, sep=".")
    assign(paste("ggp", outer.title, sep="."), value=p)
    ggsave(paste(outer.title, ".pdf", sep=""), device=cairo_pdf, plot=p,
dpi=300, width=15/2.54, height=22.8/2.54)
    ggsave(paste(outer.title, ".eps", sep=""), device=cairo_ps, plot=p,
dpi=300, width=15/2.54, height=22.8/2.54)
    ggsave(paste(outer.title, ".png", sep=""), type="cairo", plot=p,
dpi=300, width=15/2.54, height=22.8/2.54)

    # save as the enhanced metafile
    emf(file=paste(outer.title, ".emf", sep=""), width=15/2.54,
height=22.8/2.54)
    print(p)
    dev.off()

    i <- (i+1)%%2
}
warnings()

# combine pdf files
library(pdftools)
pdf_combine(paste("Fig", caption, sessions, "pdf", sep="."), output=paste
("Fig", caption, "combined", "pdf", sep="."))

# Figure files for submission
file.copy(from="Fig.success.Beta_all.eps", to="FigureS1.eps",
overwrite=TRUE, copy.date=TRUE)
file.copy(from="Fig.success.Beta_all.pdf", to="FigureS1.pdf",
overwrite=TRUE, copy.date=TRUE)
file.copy(from="Fig.success.Beta_all.emf", to="FigureS1.emf",
overwrite=TRUE, copy.date=TRUE)
file.copy(from="Fig.success.Gamma_all.eps", to="FigureS2.eps",
overwrite=TRUE, copy.date=TRUE)
file.copy(from="Fig.success.Gamma_all.pdf", to="FigureS2.pdf",
overwrite=TRUE, copy.date=TRUE)
file.copy(from="Fig.success.Gamma_all.emf", to="FigureS2.emf",
overwrite=TRUE, copy.date=TRUE)
```

Estimation accuracy of K (Figure S8)

```
caption <- "est_K"
sessions.label <- c(Beta_all="A: Estimation of k assuming Beta(mk, (n-m)k)",
                    Gamma_all="B: Estimation of k assuming gamma
distributions")
sessions <- c("Beta_all", "Gamma_all")
for (k in sessions) {
    dat.bind <- paste("bind", k, sep=".") %>% get() %>%
        dplyr::filter(ntotal %in% c(128))
    p <-ggplot(dat.bind) +
        geom_hline( yintercept=c(seq(0.01, 0.1, by=0.01), seq(0.1, 1,
by=0.1), seq(1, 10, by=1), seq(10, 100, by=10)),
                    linetype=1, color=gray(0.75), size=0.05) +
        geom_hline( yintercept=c(1), linetype=1, size=0.3 ) +
        geom_boxplot(   aes(y=K_upr/K, x=K, group=K), notch=FALSE,
                        color=hsv(0.18, 0.99, 0.1), fill=hsv(0.18, 0.99,
0.99), outlier.shape=NA, size=0.25,
                        width=1.7/length(unique(dat.bind$npertrap)),
fatten=1.5    ) + # fatten: width of median
        geom_boxplot(   aes(y=K_lwr/K, x=K, group=K), notch=FALSE,
                        color=hsv(0.58, 0.99, 0.25), fill=hsv(0.53, 0.9,
0.99), outlier.shape=NA, size=0.2,
                        width=1.5/length(unique(dat.bind$npertrap)),
fatten=1.5    ) +
        geom_boxplot(   aes(y=K_est/K, x=K, group=K), notch=FALSE,
                        color=hsv(0.5, 0, 0.15), fill=hsv(0.5, 0, 1),
outlier.shape=NA, size=0.2,
                        width=1.0/length(unique(dat.bind$npertrap)),
fatten=1.5    ) +
        facet_grid(P ~ ntotal+ntrap, margins=F, labeller=purrr::partial
(label_both, sep=" = ")) +
        theme_classic(base_size=9) +
        theme(  panel.background=element_blank(),
plot.background=element_rect(colour=NA, fill=NA),
                plot.margin=unit(c(0.25, 0.25, 0.25, 0.25), "lines")  ) + #
top, right, bottom, left
        theme(  text=element_text(family="Arial"),# plot.title=element_blank
(),
                plot.title=element_text(family="Arial", face="bold", size=8,
angle=0, hjust=0),
                strip.background=element_blank(),
                strip.text=element_text(family="Arial Narrow", face="bold",
size=8, margin=margin(b=2, t=1, l=4, r=4, unit="pt")),
                axis.text.x=element_text(family="Arial Narrow", size=6,
angle=0, hjust=0.5),
```

```
                axis.text.y=element_text(family="Arial Narrow", size=6,
angle=0, hjust=1)  ) +
        theme(  axis.line=element_line(size=0.3, color="black"),
                axis.ticks=element_line(size=0.3, color="black") ,
                axis.ticks.length=unit(0.5, "mm")  ) +
        theme(legend.position="none") +
        scale_x_log10( breaks=c(1, 3, 9, 27), labels=c(1, 3, 9, 27)) +
        scale_y_log10( limit=c(0.001, 1000), breaks=c(0.01, 0.1, 1, 10,
100), labels=c(0.01, 0.1, 1, 10, 100)) +
        labs(title=sessions.label[k], x="Real size of k", y="Estimated k /
Real k")
    outer.title <- paste("Fig", caption, k, sep=".")
    assign(paste("ggp", outer.title, sep="."), value=p)
    ggsave(paste(outer.title, ".pdf", sep=""), device=cairo_pdf, plot=p,
dpi=300, width=11/2.54, height=12/2.54)
    ggsave(paste(outer.title, ".eps", sep=""), device=cairo_ps, plot=p,
dpi=300, width=11/2.54, height=12/2.54)
    ggsave(paste(outer.title, ".png", sep=""), type="cairo", plot=p,
dpi=300, width=11/2.54, height=12/2.54)
    # save as the enhanced metafile
    emf(file=paste(outer.title, ".emf", sep=""), width=11/2.54,
height=12/2.54)
    print(p)
    dev.off()
}
warnings()

# combine pdf files
library(pdftools)
pdf_combine(paste("Fig", caption, sessions, "pdf", sep="."), output=paste
("Fig", caption, "combined", "pdf", sep="."))

# Making compound figure via cowplot
library(cowplot)
ps2 <- cowplot::plot_grid(  get(paste("ggp.Fig.est_K", sessions[1],
sep=".")),
                            get(paste("ggp.Fig.est_K", sessions[2],
sep=".")),
                            align="v", rel_heights=c(1, 1), scale=c(1, 1),
ncol=1  )
outer.title <- "Fig.est_K"
ggsave(paste(outer.title, ".pdf", sep=""), device=cairo_pdf, plot=ps2,
dpi=300, width=11.4/2.54, height=24/2.54)
ggsave(paste(outer.title, ".eps", sep=""), device=cairo_ps, plot=p, dpi=300,
width=11.4/2.54, height=24/2.54)
ggsave(paste(outer.title, ".png", sep=""), type="cairo", plot=p, dpi=300,
width=11.4/2.54, height=24/2.54)
```

```
emf(file=paste(outer.title, ".emf", sep=""), width=11.4/2.54,
height=24/2.54)
print(ps2)
dev.off()

# Figure files for submission
file.copy(from="Fig.est_K.eps", to="FigureS8.eps", overwrite=TRUE,
copy.date=TRUE)
file.copy(from="Fig.est_K.pdf", to="FigureS8.pdf", overwrite=TRUE,
copy.date=TRUE)
file.copy(from="Fig.est_K.emf", to="FigureS8.emf", overwrite=TRUE,
copy.date=TRUE)
```

Quantile of the estimated allele frequencies

ESM1 summary table: Worksheet "Simulation_Result_Beta_all"

```
Beta_all.median <- bind.Beta_all %>%
    dplyr::arrange(LID) %>%
    dplyr::group_by(P, K, ntrap, npertrap, ntotal, diploid, targetScale,
sdMeasure) %>%
    tidyr::nest() %>%
    dplyr::mutate(  zeroci=binom.test(0, as.integer(ntotal))$conf.int[2]  )
%>%
    dplyr::mutate(  used.binom=P>=zeroci,
                    used.three=ntotal>=3.0/P,
                    success.P_lwr=purrr::map_dbl(.x=data, .f=~{sum(!is.na
(.$P_lwr))/length(.$P_lwr)} %>% round(2)),
                    success.K_lwr=purrr::map_dbl(.x=data, .f=~{sum(!is.na
(.$K_lwr))/length(.$K_lwr)} %>% round(2)),
                    success.T_lwr=purrr::map_dbl(.x=data, .f=~{sum(!is.na
(.$T_lwr))/length(.$T_lwr)} %>% round(2)),
                    success.C_lwr=purrr::map_dbl(.x=data, .f=~{sum(!is.na
(.$C_lwr))/length(.$C_lwr)} %>% round(2)),
                    median.P_lwr=purrr::map_dbl(.x=data, .f=~median(.$P_lwr,
na.rm=TRUE)),
                    median.P_upr=purrr::map_dbl(.x=data, .f=~median(.$P_upr,
na.rm=TRUE)),
                    median.K_lwr=purrr::map_dbl(.x=data, .f=~median(.$K_lwr,
na.rm=TRUE)),
                    median.K_upr=purrr::map_dbl(.x=data, .f=~median(.$K_upr,
```

```
na.rm=TRUE)),
                    median.T_lwr=purrr::map_dbl(.x=data, .f=~median(.$T_lwr,
na.rm=TRUE)),
                    median.T_upr=purrr::map_dbl(.x=data, .f=~median(.$T_upr,
na.rm=TRUE)),
                    median.C_lwr=purrr::map_dbl(.x=data, .f=~median(.$C_lwr,
na.rm=TRUE)),
                    median.C_upr=purrr::map_dbl(.x=data, .f=~median(.$C_upr,
na.rm=TRUE))  )
Beta_all.median <- Beta_all.median %>%
    dplyr::select(-zeroci, -data) %>%
    dplyr::select(diploid, K, P, ntotal, ntrap, npertrap, everything()) %>%
    dplyr::ungroup() %>%
    dplyr::arrange(K, P, ntotal, ntrap) %>%
    dplyr::mutate(  Width.P_lwr=round(P/median.P_lwr, 2),
                    Width.P_upr=round(median.P_upr/P, 2),
                    Width.K_lwr=round(K/median.K_lwr, 2),
                    Width.K_upr=round(median.K_upr/K, 2),
                    Width.T_lwr=round(targetScale/median.T_lwr, 2),
                    Width.T_upr=round(median.T_upr/targetScale, 2),
                    Width.C_lwr=round(sdMeasure/median.C_lwr, 2),
                    Width.C_upr=round(median.C_upr/sdMeasure, 2),  )

summary(Beta_all.median)
readr::write_csv(Beta_all.median, "Beta_all.median.csv")
```

ESM1 summary table: Worksheet "Simulation_Result_Beta_K1"

```
Beta_K1.median <- bind.Beta_K1 %>%
    dplyr::arrange(LID) %>%
    dplyr::group_by(P, K, ntrap, npertrap, ntotal, diploid, targetScale,
sdMeasure) %>%
    tidyr::nest() %>%
    dplyr::mutate(  zeroci=binom.test(0, as.integer(ntotal))$conf.int[2]  )
%>%
    dplyr::mutate(  used.binom=P>=zeroci,
                    used.three=ntotal>=3.0/P,
                    success.P_lwr=purrr::map_dbl(.x=data, .f=~{sum(!is.na
(.$P_lwr))/length(.$P_lwr)} %>% round(2)),
                    success.K_lwr=purrr::map_dbl(.x=data, .f=~{sum(!is.na
(.$K_lwr))/length(.$K_lwr)} %>% round(2)),
                    success.T_lwr=purrr::map_dbl(.x=data, .f=~{sum(!is.na
(.$T_lwr))/length(.$T_lwr)} %>% round(2)),
```

```
                        success.C_lwr=purrr::map_dbl(.x=data, .f=~{sum(!is.na
(.$C_lwr))/length(.$C_lwr)} %>% round(2)),
                        median.P_lwr=purrr::map_dbl(.x=data, .f=~median(.$P_lwr,
na.rm=TRUE)),
                        median.P_upr=purrr::map_dbl(.x=data, .f=~median(.$P_upr,
na.rm=TRUE)),
                        median.K_lwr=purrr::map_dbl(.x=data, .f=~median(.$K_lwr,
na.rm=TRUE)),
                        median.K_upr=purrr::map_dbl(.x=data, .f=~median(.$K_upr,
na.rm=TRUE)),
                        median.T_lwr=purrr::map_dbl(.x=data, .f=~median(.$T_lwr,
na.rm=TRUE)),
                        median.T_upr=purrr::map_dbl(.x=data, .f=~median(.$T_upr,
na.rm=TRUE)),
                        median.C_lwr=purrr::map_dbl(.x=data, .f=~median(.$C_lwr,
na.rm=TRUE)),
                        median.C_upr=purrr::map_dbl(.x=data, .f=~median(.$C_upr,
na.rm=TRUE))  )
Beta_K1.median <- Beta_K1.median %>%
    dplyr::select(-zeroci, -data) %>%
    dplyr::select(diploid, K, P, ntotal, ntrap, npertrap, everything()) %>%
    dplyr::ungroup() %>%
    dplyr::arrange(K, P, ntotal, ntrap) %>%
    dplyr::mutate(  Width.P_lwr=round(P/median.P_lwr, 2),
                    Width.P_upr=round(median.P_upr/P, 2),
                    Width.K_lwr=round(K/median.K_lwr, 2),
                    Width.K_upr=round(median.K_upr/K, 2),
                    Width.T_lwr=round(targetScale/median.T_lwr, 2),
                    Width.T_upr=round(median.T_upr/targetScale, 2),
                    Width.C_lwr=round(sdMeasure/median.C_lwr, 2),
                    Width.C_upr=round(median.C_upr/sdMeasure, 2),   )

summary(Beta_K1.median)
readr::write_csv(Beta_K1.median, "Beta_K1.median.csv")
```
